# Extracellular vesicles enriched with mitochondrial components from intermittently cold-exposed adipose tissue drive metabolically active adipose regeneration via miR-296-3p

**DOI:** 10.1016/j.mtbio.2026.103321

**Published:** 2026-06-05

**Authors:** Shaowei Zhu, Jie Sun, Chenggang Yi, Jing Wang, Jun Yin

**Affiliations:** a The State Key Laboratory of Fluid Power and Mechatronic Systems, School of Mechanical Engineering, Zhejiang University, Hangzhou, 310027, China; b Department of Plastic and Reconstructive Surgery, The First Affiliated Hospital of Ningbo University, Ningbo, 315010, China; c Center for Medical and Engineering Innovation, Central Laboratory, The First Affiliated Hospital of Ningbo University, Ningbo, 315010, China; d Department of Neurosurgery, Ningbo Key Laboratory of Nervous System and Brain Function, The First Affiliated Hospital of Ningbo University, Ningbo, Zhejiang, 315010, China; e Department of Plastic Surgery, The Second Affiliated Hospital of Zhejiang University College of Medicine, Hangzhou, 310000, China

**Keywords:** Acellular adipose matrix, Decellularized adipose tissue, Adipose tissue engineering, Intermittent cold exposure, Extracellular vesicles, Metabolic reprogramming

## Abstract

Current acellular adipose tissue engineering focuses primarily on structural regeneration, whereas functional metabolic recovery remains a major challenge. Extracellular vesicles (EV) derived from adipose tissue (AT-EV) have recently emerged as potent mediators of tissue regeneration, and cold stimulation has been reported to enhance the metabolic reprogramming of AT-EV. We have previously reported that fat grafts from cold-stimulated mice exhibited enhanced survival and adipose regeneration. Yet whether cold-induced AT-EV contribute to in vivo metabolic adipose regeneration remains unclear. Moreover, the cold stimulation temperatures used in traditional mouse models are often intolerable for humans. To address this, we developed a short-term intermittent mild cold exposure (STIMCE) protocol to metabolically activate murine adipose tissue for EV isolation (STIMCE-EV). Following only 3 or 7 days of STIMCE treatment, adipose tissue exhibited increased EV secretion and enrichment of mitochondrial components. Moreover, STIMCE-EV were efficiently incorporated into human acellular adipose matrix (AAM) hydrogel and exhibited controlled release in vitro. Injectable AAM@STIMCE-EV hydrogel transplantation in nude mice showed improved adipogenesis and volume retention in situ after 2 months. Importantly, the AAM@STIMCE-EV grafts improved cold adaptation and enhanced the thermogenic function of the host mice. Mechanistically, mmu-miR-296-3p was identified as a key regulatory factor within STIMCE-EV. Exogenous mmu-miR-296-3p supplementation significantly improved the adipogenic regeneration of AAM. Overall, this study presents a metabolically activated, cell-free adipose regeneration strategy that combines STIMCE-EV with AAM scaffolds. This minimally invasive and injectable system enables functional adipose tissue regeneration with systemic thermogenic metabolic activity, providing a promising translational solution for soft tissue reconstruction.

## Highlights

• STIMCE metabolically activates adipose extracellular vesicles.
• STIMCE-EV are enriched with mitochondrial components.
• Injectable AAM@STIMCE-EV hydrogel promotes efficient adipose tissue regeneration.
• AAM@STIMCE-EV grafts enhance thermogenesis in host mice under cold stress.
• miR-296-3p is a key mediator of STIMCE-EV that promotes the adipose regeneration.

## Graphical abstract

**Figure:**
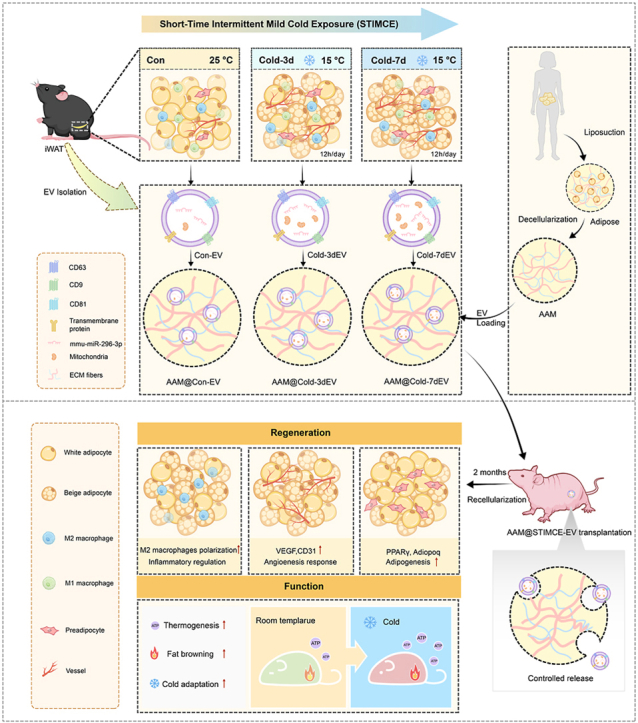

## 1 Introduction

Adipose tissue not only serves as an energy reservoir but also modulates thermogenesis, hormonal balance, and immune responses [[1], [2], [3]]. Fat defects resulting from disease or clinical conditions such as malformation, trauma, oncologic resection, and hemifacial atrophy cause aesthetic and functional disorders [4,5]. Autologous fat grafting is considered the gold standard for adipose tissue reconstruction due to its excellent biocompatibility and natural integration [[6], [7], [8]]. However, patients with low body mass index (BMI), cancer cachexia, or lipodystrophy often lack sufficient harvestable fat [8,9]. Hence, the limited volume of accessible autologous fat may not meet the clinical demand in reconstructive surgery for large soft tissue defects [10]. In addition, allogeneic fat transplantation may encounter difficulties associated with immune rejection and inflammatory regulation [9,10].

Compared with traditional fat grafting, natural cell-free scaffolds have emerged as an important research direction for adipose tissue regeneration with reduced immune risk [11,12]. Among them, the extracellular matrix (ECM) from decellularized tissue serves as a natural scaffold that enables cell attachment and proliferation, while gradually being remodeled and replaced by host-derived tissue when transplanted in vivo [11]. Among various decellularized ECM scaffolds, acellular adipose matrix (AAM) has been shown to spontaneously induce adipogenesis in vivo and is readily accessible clinically [[13], [14], [15], [16]]. By preserving native ECM architecture, AAM facilitates effective soft tissue reconstruction [[17], [18], [19]]. AAM is reported to survive long-term in vivo and regenerate adipocytes in nude mice [20]. Moreover, different components of AAM have been reported to contribute differently to adipose tissue regeneration [[21], [22], [23]].

Although AAM retains the native ECM architecture crucial for adipocyte differentiation, complete decellularization compromises its regenerative and metabolic capacities due to the lack of important cells and bioactive factors [24,25]. Thus, AAM alone may show limited adipogenesis and vascularization [14,26]. Extracellular vesicles (EV) secreted from adipose tissue, also referred to as adipose tissue-derived EV (AT-EV), have attracted increasing interest since they can act as intercellular messengers [27,28]. AT-EV have been shown to promote proliferation and differentiation of adipose-derived stromal cells (ADSCs), as well as to improve vascular regeneration, and polarize macrophages towards a regenerative phenotype [13,29]. While these properties render them appealing components for cell-free adipose regenerative strategies, the use of EV alone is limited by rapid clearance, uncontrolled diffusion, and a lack of sustained release, which hinder their effective in vivo performance [13]. Incorporating EV into suitable scaffolds to achieve controlled release represents a viable approach for tissue reconstruction [30]. AAM scaffolds possess a loose and porous ECM structure, which enables EV loading and controlled release [17,26]. The AAM@EV hybrid scaffold, as an enhanced cell-free strategy, demonstrates significant efficacy in promoting in vivo tissue remodeling [29,31].

Numerous studies have focused on the structural reconstruction of adipose tissue, whereas the recovery of metabolic function has received little attention [4]. Adipose tissue is metabolically active, thermogenic, and sensitive to cold exposure [32,33]. The restoration of thermogenic capacity and systemic metabolic activity is important for functional adipose tissue regeneration [26]. Clinical studies indicate that cold acclimatization mitigates insulin resistance in individuals with type 2 diabetes, while also promoting metabolic health in the obese population [32,34]. Cold stimulation can induce increased mitochondrial content and the upregulation of thermogenic genes [35,36]. Moreover, cold-induced adipose reprogramming also modifies the composition and biological function of AT-EV, which may be enriched with thermogenic signaling-related components [37]. Cold-stimulated AT-EV have been reported to exhibit unique metabolic functions, including the stimulation of hepatic gluconeogenesis and the promotion of weight loss in high-fat diet mice [37]. We previously reported that the browning of white adipocytes enhanced skin wound healing and improved the survival of adipose grafts [[38], [39], [40]]. However, it remains unclear whether EV derived from cold-stimulated adipose tissue can contribute to the engineering of metabolically functional adipose tissue.

Most studies involving cold stimulation in animals are performed at 4 °C, which may not be tolerable for humans [32]. Recent findings have revealed that even short-term cold exposure can establish a long-lasting epigenomic memory in adipose tissue [41]. We have previously reported that cold exposure prior to transplantation significantly improves fat graft survival and regeneration [42]. Here, we hypothesized that EV derived from mild cold-stimulated adipose tissue could contribute to the construction of metabolically functional adipose tissue. To test our hypothesis, we established a model in which mice were subjected to short-term intermittent mild cold exposure (STIMCE). We then isolated AT-EV derived from inguinal white adipose tissue (iWAT) as STIMCE-EV and prepared AAM from human subcutaneous adipose tissue. In this study, we employed AAM loaded with STIMCE-EV for adipose tissue regeneration in nude mice, establishing an enhanced functional cell-free adipose regeneration approach. Furthermore, we confirmed that STIMCE-EV promoted significantly greater AAM-mediated adipose regeneration than control AT-EV. Importantly, STIMCE-EV promoted cold tolerance and thermogenic metabolic activity of the host mice under cold stimulation at 15 °C. Overall, these results provide a new perspective for the advancement of cell-free adipose regeneration strategies and offer a new direction for achieving metabolically active and functional adipose tissue.

## 2 Materials and methods

### 2.1 Sample collection and ethical statement

Human subcutaneous adipose tissue was harvested from healthy female donors undergoing elective liposuction procedures (age, 32.75 ± 5.56 years; BMI, 21.1 ± 1.4 kg/m^2^). Sample collection was approved by the Ethics Committee of the Second Affiliated Hospital, Zhejiang University School of Medicine (NO. 20230415).

### 2.2 Preparation of AAM hydrogel

The enzyme-free decellularization method was performed as previously reported [23,43]. In brief, purified adipose tissue was homogenized and then centrifuged at 3500 × *g* for 3 min and centrifuged at 3000 × *g* for 3 min. The oil layer, aqueous phase, and pellet were removed, and the layer below the oil was collected as the pre-AAM fraction. The material was then sequentially treated with 1 M NaCl (6 h), ddH_2_O (10 h) and 1% Triton X-100 (48 h) solutions respectively. Afterward, the samples were washed three times with sterile distilled water (for 0.5 h each), immersed in 99% isopropanol for 6 h to remove residual lipids, followed by an additional rinse with sterile water. Finally, the processed AAM was sterilized three times with 75% ethanol and lyophilized at −80 °C.

For hydrogel formation, the lyophilized AAM was ground into a powder and dissolved in a 0.01 M HCl solution containing 1 mg/mL pepsin to achieve a final AAM concentration of 10 mg/mL. The mixture was digested at room temperature under magnetic stirring at 60 rpm for 48 h until a homogeneous liquid with no macroscopic particles was observed. Subsequently, the pH was adjusted to 7.4 using a 0.1 M NaOH solution, and the osmotic pressure was balanced using a 10× PBS solution. All steps were performed on ice. Finally, the pre-gel AAM solution was incubated at 37 °C for 30 min to induce spontaneous gelation.

### 2.3 Identification and evaluation of AAM

Quantification of residual DNA was carried out to assess decellularization efficiency. Approximately 5 mg of lyophilized AAM was digested in 10 mg/mL proteinase K solution at 55 °C until complete tissue dissolution. Genomic DNA was extracted (Solarbio, Beijing, China) and quantified using a NanoDrop 2000 (Thermo Fisher Scientific, USA). Final concentrations were adjusted based on the dry weight of the tissue (ng DNA/mg AAM). A residual DNA content lower than 50 ng/mg was considered indicative of adequate decellularization quality.

To evaluate the retention of intrinsic bioactive molecules, growth factor (EGF, PDGF-BB, and bFGF) levels were analyzed using a quantitative multiplex Luminex® assay kit (LXSAHM-04, R&D Systems®, Minnesota, USA) according to the manufacturer's instructions as previously reported. Briefly, 50 μL of appropriately diluted samples were incubated with biotinylated antibody and PE-conjugated streptavidin. Each sample was analyzed in triplicate using a flow cytometer (BD LSRFortessa SORP, USA).

### 2.4 STIMCE model

All animal experiments were approved by the Animal Ethics Committee of the Second Affiliated Hospital of Zhejiang University School of Medicine (Approval No. AIRB-2023-0983). Mice were housed in a specific pathogen-free (SPF) facility where environmental parameters (temperature and humidity) were strictly controlled. The animals followed a standard 12-h photoperiod, with unrestricted access to chow and water. The establishment of the STIMCE model involved maintaining 8-week-old male C57BL/6J mice in specifically regulated chambers (Powers Scientific, R1S33D) under controlled climatic conditions. Mice were singly housed and randomly divided into three groups: Control (Con), Cold-3d, and Cold-7d. Control mice were maintained continuously at standard room temperature (RT, 25 ± 1 °C) with a 12 h light/12 h dark cycle. For the STIMCE groups, mice were subjected to alternating cycles of RT (25 °C for 12 h) and mild cold (15 °C for 12 h) in a temperature-controlled climatic chamber for either 3 consecutive days (Cold-3d) or 7 consecutive days (Cold-7d). Upon completion of the exposure phase, the mice were anesthetized and subsequently sacrificed, and inguinal white adipose tissue was harvested for EV isolation as well as histological and molecular assays.

### 2.5 Isolation and identification of EV from iWAT

EV isolation followed previously established protocols using a stepwise ultracentrifugation procedure [44]. In brief, freshly harvested iWAT from Con, Cold-3d, and Cold-7d mice was carefully minced into fragments (<3 mm^3^) and digested with 1% collagenase I (37 °C, 40 min). Digestion was halted by introducing an equivalent volume of PBS containing 2 mmol/L EGTA. The suspension was clarified via sequential centrifugation at 4 °C: initially at 300 × g (10 min), followed by 2000 × g (20 min) and 10,000 × g (30 min) to deplete cells and debris. Subsequently, the supernatant was filtered (0.22 μm), loaded into Beckman Coulter tubes (#355618), and ultracentrifuged at 100,000 × g for 70 min to pellet the EV.

Immunoblotting was conducted on EV markers to confirm the expression of CD9 and CD63. The particle size distribution and concentration of EV were measured using a NanoSight NS300 (Malvern Panalytical, UK). Each EV suspension was diluted with sterile PBS before measurement. Samples from Con-EV, Cold-3dEV, and Cold-7dEV were injected at the same flow rate and recorded at the same time. Video data were analyzed to calculate the mean particle diameter and particle concentration. We utilized transmission electron microscopy (TEM) to characterize the morphology and ultrastructure of Con-EV, Cold-3dEV, and Cold-7dEV. Sample loading involved placing 20 μL aliquots onto Formvar-carbon grids (200 mesh) for 2 min. Following a distilled water rinse, negative staining was carried out using 1% uranyl acetate for 1 min. The grids were then air-dried and micrographs were captured via a FEI Tecnai G2 Spirit instrument operating at 120 kV.

### 2.6 Preparation and controlled release analysis of AAM@EV hydrogel

The Con-EV, Cold-3dEV, and Cold-7dEV suspensions were independently mixed with the pre-gel AAM solution to achieve a final EV concentration of 100 μg/mL for each group. For the evaluation of the release profiles, 500 μL of each respective AAM@EV hydrogel (AAM@Con-EV, AAM@Cold-3dEV, or AAM@Cold-7dEV) solution was added into the upper chamber of a 24-well Transwell insert and incubated at 37 °C to allow for gelation. Subsequently, 1 mL of PBS was added to the lower chamber. At predetermined time intervals (days 1, 2, 3, 4, 5, 7, 9, and 11), a 50 μL aliquot of the release medium was collected and replenished with fresh PBS. The released EV protein content was quantified using a BCA assay kit to generate the cumulative release profiles.

### 2.7 Cellular internalization assay

To examine cellular uptake, EV were labeled with the fluorescent dye DiI (cat#C7000, Invitrogen, USA) and then loaded into AAM hydrogel as described above. AAM@DiI-EV were cocultured with human stromal vascular fraction (hSVF) cells and human umbilical vein endothelial cells (HUVECs) for 24 h at 37 °C using a Transwell system (Corning Costar, 0.4 μm pore size). Post-incubation, the cells were rinsed with PBS and subjected to fixation in 4% paraformaldehyde for 15 min. Nuclear counterstaining was performed using DAPI. Finally, images of the fluorescence signals from the DiI-labeled EV were acquired using a confocal laser scanning microscope (Carl Zeiss, Germany).

### 2.8 In vitro coculture model

A Transwell system (Thermo Scientific, 0.4 μm pore size) was used to allow the exchange of EV and soluble factors between the upper and lower chambers. The AAM, AAM@Con-EV, AAM@Cold-3dEV, and AAM@Cold-7dEV hydrogels were independently placed into the upper chambers, while HUVECs or hSVF cells were cultured in the lower chamber. The control group (Con) contained empty inserts without any hydrogel. For HUVECs, after 48 h of coculture, the cells were subjected to Ki67 staining and tube formation assays. For hSVF cells, after 72 h of coculture, cell viability was evaluated using a CCK-8 assay. Additionally, cells cocultured for 48 h were harvested for subsequent analyses.

### 2.9 Isolation and culture of hSVF cells

Blood contaminants were removed via triple PBS washing and centrifugation (300 × g, 5 min). The adipose tissue was then incubated with 0.1% collagenase type I (in DMEM, 37 °C) and agitated gently for 40 min until a homogeneous suspension was achieved. To quench the digestion, serum-containing medium (DMEM + 10% FBS + 1% antibiotics) was added at a 1:1 ratio. To eliminate tissue debris, the suspension was passed through a 70 μm mesh. Following centrifugation (600 × g, 10 min), the supernatant was aspirated, and the resulting pellet (containing primarily hSVF cells) was resuspended in fresh medium. The Human Adipose Mesenchymal Stem Cell Adipogenic Induction Differentiation Kit (cat# HUXMD-90031, OriCell) and the Human Adipose Mesenchymal Stem Cell Osteogenic Induction Differentiation Kit (cat# HUXMD-90021, OriCell) were used to evaluate the multipotent differentiation ability of hSVF cells.

### 2.10 Scratch assay

Following the establishment of a 100% confluent HUVEC monolayer in the lower chambers, a standardized scratch was generated using a 200 μL tip. Detached cells were washed away with PBS. The lower chambers were then coupled with their respective inserts for a 24-h coculture period at 37 °C (5% CO_2_). Wound closure was quantified by capturing images at 0 h and 24 h, and the migration rate was determined using ImageJ software as a percentage of the initial scratch width.

### 2.11 Tube formation assay

Cocultured HUVECs were detached with 0.25% trypsin-EDTA (cat#25200056, Thermo Fisher, USA) and resuspended at 1 × 10^4^ cells/mL. Matrigel (Corning 354234) was thawed on ice overnight, 50 μL was added per well in a prechilled 96-well plate, and polymerized at 37 °C for 30 min. Then, 100 μL of cocultured HUVEC suspension was seeded on the Matrigel and incubated for 6 h. Tube structures were photographed and total tube length and number of junctions were quantified using ImageJ software.

### 2.12 CCK-8 assay

hSVF cells were seeded in the lower chamber of a 24-well Transwell plate (cat#140620, Thermo Scientific) at a density of 1 × 10^4^ cells/well. After 72 h of coculture, the Transwell plates were removed from the incubator. CCK-8 solution (cat#C0038, Beyotime) was added and incubated at 37 °C for 2 h, and absorbance was recorded at 450 nm.

### 2.13 Seahorse assay

The AAM or AAM@EV hydrogel was prepared in the upper chamber of a Transwell insert. Complete cell culture medium was added to the lower chamber. After 48 h of incubation at 37 °C, the conditioned medium in the lower chamber was collected for subsequent cell treatments. hSVF cells (8000 cells/well) were plated in Seahorse XF24 cell culture microplates (Agilent Technologies) and cultured overnight to allow attachment. The culture medium was then replaced with the collected CM from different groups (Con, AAM, AAM@Con-EV, AAM@Cold-3dEV, and AAM@Cold-7dEV). Mitochondrial function was assessed using the Seahorse XF Mito Stress Test (Agilent), involving serial injections of oligomycin (2 μM), FCCP (1.2 μM), and rotenone/antimycin A (1 μM). Key metrics (basal respiration, ATP production, and maximal respiration capacity) were calculated after background correction and standardized against protein content (BCA assay, Beyotime).

### 2.14 Western blot analysis

Samples were solubilized in chilled RIPA buffer (cat# 89900, Invitrogen) fortified with a protease and phosphatase inhibitor cocktail (cat# 78440, Invitrogen). Subsequently, protein levels were quantified using the Pierce BCA assay (cat# 23227, Invitrogen). The samples were resolved by SDS-PAGE and the target antigens were detected using the following antibodies: anti-UCP1 (1:1000, cat# PA1-24894, Invitrogen), anti-PGC1α (1:1000, cat# MA5-32563, Invitrogen), anti-CD9 (1:1000, cat# MA5-31980, Invitrogen), anti-CD63 (1:1000, cat# ab217345, Abcam), anti-F4/80 (1:1000, cat# 14-4801-82, Invitrogen), anti-CD206 (1:500, cat# PA5-101657, Invitrogen), and anti-SOCS6 (1:1000, cat# ab197335, Abcam). Band intensities were analyzed using ImageJ software.

### 2.15 Immunofluorescence staining

To label the mitochondrial network, hSVF cells were incubated with 100 nM MitoTracker (cat# M22425, Invitrogen) for 30 min at 37 °C. Following a PBS rinse, the cells were fixed with 4% paraformaldehyde and subsequently counterstained with DAPI to mark the nuclei (cat# D1306, Invitrogen), and imaged by confocal microscopy (Zeiss LSM 880, Germany). Immunofluorescence staining was performed on both cultured cells (HUVECs and hSVF cells) and tissue sections. The cells were rinsed with PBS and subjected to fixation using 4% paraformaldehyde (15 min). Subsequently, permeabilization was performed with 0.1% Triton X-100, followed by a blocking step using 3% BSA for 1 h at ambient temperature. Primary antibodies were incubated overnight at 4 °C using the following reagents and dilutions: anti-UCP1 (1:500, cat# PA1-24894, Invitrogen), and anti-Ki67 (1:200, cat# MA5-14520, Invitrogen). For tissue sections (5 μm), after deparaffinization and antigen retrieval (10 mM, cat# 00-4955-58, Invitrogen), the samples were then incubated with the specific primary antibodies overnight at 4 °C, as follows: anti-Perilipin1 (1:200, cat# 9349, Abcam), anti-F4/80 (1:100, cat# MA5-16363, Invitrogen), and anti-CD206 (1:200, cat# ab300621, Abcam). Sections were then incubated with Alexa Fluor 488 (1:200, cat# A-11008, Invitrogen) or Alexa Fluor 594 (1:200, cat# A-11012, Invitrogen) for 1 h. After washing, DAPI (cat# D1306, Invitrogen) was applied for 15 min, and samples were visualized using a Zeiss LSM 880 confocal microscope, followed by image analysis via ImageJ.

### 2.16 RNA isolation and real-time quantitative PCR (qRT-PCR)

Total RNA was extracted from cultured cells or tissues using the PureLink™ RNA Microextraction Kit (cat#12183016, Invitrogen). RNA concentration was determined with a NanoDrop 8000 (Thermo, USA). A total of 1 μg of RNA was used to synthesize cDNA using SuperScript™ II Reverse Transcriptase (cat#18064014, Invitrogen). Real-time quantitative PCR was conducted with a LightCycler®480 (Roche Diagnostics, Mannheim, Germany using PowerTrack™SYBR Green Master Mix (cat#A46110, Invitrogen). The threshold cycle (Ct) values were acquired and analyzed using the 2^−ΔΔCt^ method. The target sequences are listed in Supplementary Table 1.

### 2.17 AAM implantation

Eight-week-old male BALB/c nude mice (GemPharmatech Laboratory, Nanjing, China) were randomly divided into four groups: AAM, AAM@Con-EV, AAM@Cold-3dEV, and AAM@Cold-7dEV (n = 12). Briefly, 100 μL of AAM or AAM@EV hydrogel was subcutaneously injected into the dorsal region of each mouse using an 18-gauge needle. Two symmetrical injection sites were made approximately 1 cm lateral to the midline on each side of the back. Grafts were harvested at 1 and 2 months after implantation for morphological, histological, and molecular analyses. For routine hematology and serum biochemical assays, blood was drawn following animal termination. To evaluate tissue morphology, the major visceral organs—specifically the heart, liver, spleen, lung, and kidney—were harvested and subjected to hematoxylin and eosin (H&E) staining.

### 2.18 Transcriptomic sequencing and analysis

Total RNA was harvested from the graft samples (AAM@Cold-7dEV, n = 4 vs. AAM@Con-EV, n = 5), with integrity confirmed (RIN > 7.0) via an Agilent 2100 Bioanalyzer. Sequencing libraries were constructed using the Hieff NGS® Ultima Dual-mode kit (Yeasen, #12252) and subjected to 150-bp paired-end sequencing on a DNBSEQ-T7 system. Post-filtering, reads were mapped to the murine genome (mm10) utilizing HISAT2, followed by quantification via featureCounts. Differential expression analysis was carried out using edgeR (|log_2_FC| > 1, P < 0.05). Finally, functional annotation (GO, KEGG) and enrichment (GSEA) were executed with the clusterProfiler R package. Visualization and annotation were performed using ggplot2.

### 2.19 Small RNA sequencing and analysis

Small RNA profiling was conducted on the Cold-7dEV and Con-EV groups (3 biological replicates each) utilizing the DNBSEQ-T7 Sequencing Platform. Prior to sequencing, RNA purification was achieved via the MirVana miRNA Isolation Kit (cat#AM1561, Thermo Fisher Scientific), and sequencing libraries were generated using the VAHTS Small RNA Library Prep Kit for Illumina V2. Clean reads were processed to remove adapters and mapped to miRBase v22 for known miRNA identification. Differentially expressed miRNAs were screened using edgeR (|log_2_FC| > 1, P < 0.05). Target genes were predicted using publicly available algorithms including TargetScan, miRWalk and miRDB. Functional annotation was executed via the clusterProfiler R package to determine GO term enrichment.

### 2.20 Metabolic cage measurement

At 2 months post-implantation, mice from each group (AAM, AAM@Con-EV, AAM@Cold-3dEV, and AAM@Cold-7dEV) were individually acclimated to metabolic cages for 24 h, followed by comprehensive metabolic monitoring in a CLAMS system (Columbus Instruments, USA) (n = 6). The monitoring protocol consisted of two distinct phases: an initial 24 h period at 30 °C followed by a 24 h cold challenge at 15 °C. Concurrently, core body temperature was assessed using a BAT-12 rectal probe (Physitemp, New Jersey).

### 2.21 In vitro delivery of mmu-miR-296-3p mimic and inhibitor

mmu-miR-296-3p mimic (Mic) and inhibitor (Inh), together with negative controls (MicNC and InhNC), were synthesized for in vitro delivery (Invitrogen). hSVF cells were maintained in DMEM containing 10% FBS. The cultures were kept at 37 °C in a humidified atmosphere with 5% CO_2_. qRT-PCR was used to verify the expression level of mmu-miR-296-3p. For CCK-8 and Seahorse assays, hSVF cells that reached 60–70% confluence were transfected using Lipofectamine 3000 (cat#L3000150, Invitrogen) at 30 nM (MicNC, Mic, InhNC, and Inh) following the manufacturer's instructions.

### 2.22 In vivo delivery of mmu-miR-296-3p agomiR (AgomiR) and antagomiR (AntamiR)

mmu-miR-296-3p AgomiR and AntamiR, together with negative controls (AgoNC and AntaNC), were synthesized by Invitrogen for in vivo delivery. For the gain-of-function experiments, 100 μL of AAM hydrogel alone, or AAM hydrogel containing 4 nmol of either AgomiR-296-3p or AgoNC was transplanted in vivo as described above (n = 6). For the loss-of-function experiments, 100 μL of AAM@Cold-7dEV hydrogel alone or containing 8 nmol of either AntamiR-296-3p or AntaNC was transplanted (n = 6). The grafts were harvested 2 months post-transplantation for subsequent histological and molecular analyses.

### 2.23 Dual-luciferase reporter assay

The WT and MUT SOCS6 3′-UTR fragments containing the predicted mmu-miR-296-3p binding site were cloned into the psiCHECK-2 Vector (Cat# C8021, Promega, USA). hSVF cells were seeded in 12-well plates at a density of 1 × 105 cells per well. Cells were co-transfected with 0.2 μg of WT or MUT reporter plasmid and mmu-miR-296-3p mimic or negative control (50 nM) using Lipofectamine 3000 Transfection Reagent (Cat# L3000015, Thermo Fisher Scientific, USA). After 48 h, cells were lysed, and luciferase activities were measured using the Dual-Luciferase Reporter Assay System (Cat# E1910, Promega, USA) according to the manufacturer's instructions.

### 2.24 Statistical analysis

Data were analyzed using GraphPad Prism 10.1 software (San Diego, CA) and are presented as mean ± SEM from at least three independent experiments. For normally distributed data, comparisons were performed using an unpaired two-tailed Student's t-test, one-way ANOVA or two-way ANOVA, followed by post-hoc tests. Statistical significance was indicated as follows: *P < 0.05, **P < 0.01, and ***P < 0.001.

## 3 Results

### 3.1 STIMCE promotes thermogenic remodeling and mitochondrial biogenesis in murine iWAT

**Fig. 1:**
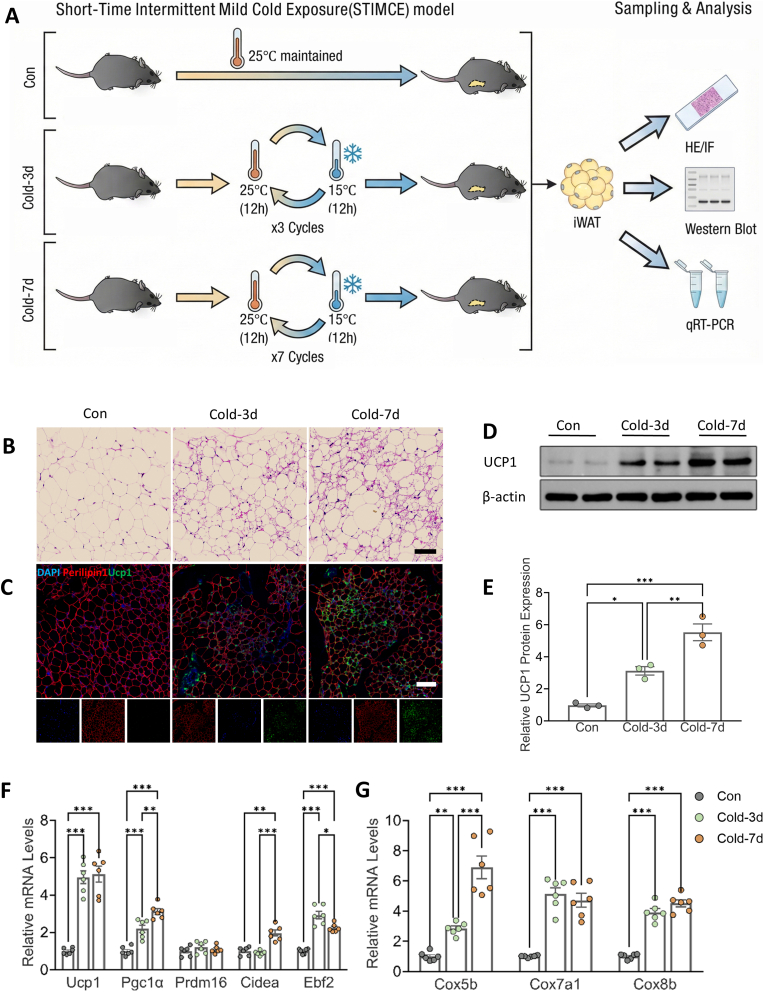
STIMCE promotes thermogenic remodeling and mitochondrial biogenesis in murine iWAT. **(A)** Schematic illustration of the STIMCE model. Eight-week-old male C57BL/6J mice were maintained at a constant 25 °C (Con) or subjected to intermittent cold stimulation (STIMCE) consisting of alternating 12 h/12 h cycles of 25 °C and 15 °C for 3 cycles (Cold-3d) or 7 cycles (Cold-7d). Subsequently, inguinal white adipose tissue (iWAT) was harvested for histological and molecular analyses. **(B)** H&E staining of iWAT. Scale bar: 50 μm. **(C)** Representative confocal immunofluorescence images of UCP1 (green) and Perilipin-1 (red) co-staining in iWAT. Scale bar: 100 μm. **(D**–**E)** Western blot analysis and corresponding quantification of UCP1 protein levels in iWAT (n = 3). **(F**–**G)** Relative mRNA expression of thermogenic-related genes (**F**) and mitochondrial-related (**G**) genes in iWAT as determined by qRT-PCR (n = 6). *P < 0.05, **P < 0.01, ***P < 0.001. (For interpretation of the references to colour in this figure legend, the reader is referred to the Web version of this article.)

To investigate the potential impact of STIMCE on the metabolic remodeling of iWAT, we established a murine model as depicted in Fig. 1A. Mice were subjected to alternating cycles of room temperature (RT, 25 °C for 12 h) and mild cold (15 °C for 12 h) for 3 or 7 consecutive days (designated as Cold-3d and Cold-7d, respectively). Control (Con) mice were kept continuously at RT. H&E staining showed that relative to the Con group, STIMCE treatment caused obvious morphological changes in iWAT, characterized by the appearance of small, multilocular lipid droplets (Fig. 1B). Notably, this phenotype was even more pronounced in the Cold-7d group, suggesting a gradual, time-dependent browning of white adipocytes. In line with these morphological changes, immunofluorescence co-staining of Perilipin-1 and UCP1 confirmed the thermogenic adaptation, revealing an efficient induction of UCP1 protein in the iWAT of both Cold-3d and Cold-7d mice (Fig. 1C). Moreover, western blotting demonstrated an increase in UCP1 protein after STIMCE, with the most prominent increase seen in the Cold-7d group (Fig. 1D and E). To better define these molecular changes at the transcriptional level, the expression of selected thermogenic and mitochondrial genes was evaluated by qRT-PCR. The results showed that compared with the Con group, STIMCE treatment significantly increased the expression of thermogenesis-associated genes, including Ucp1, Pgc1a, Prdm16, Cidea, and Ebf2 (Fig. 1F). Intriguingly, the expression of genes critical for mitochondrial oxidative phosphorylation, such as Cox5b, Cox7a1, and Cox8b, was also markedly enhanced, with their expression levels peaking in the Cold-7d group (Fig. 1G). Collectively, these findings demonstrate that STIMCE effectively drives the thermogenic program and mitochondrial activation in iWAT, establishing a foundation for subsequent investigations into the bioactive factors released during this browning process.

### 3.2 STIMCE enhances the biogenesis and mitochondrial protein enrichment of iWAT-derived EV

**Fig. 2:**
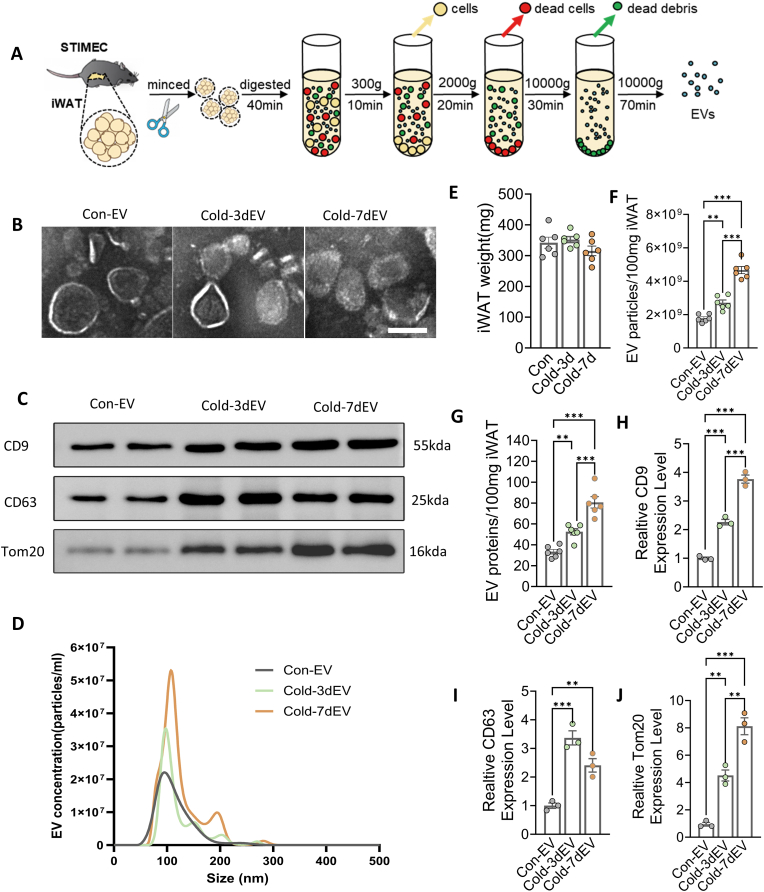
STIMCE enhances the biogenesis and mitochondrial protein enrichment of iWAT-derived EV. **(A)** Schematic diagram of the iWAT EV isolation procedure. Freshly harvested iWAT from Con, Cold-3d, and Cold-7d mice was carefully minced into small fragments and digested with 1% collagenase I. A stepwise ultracentrifugation procedure was used for EV isolation. **(B)** EV were captured by TEM. Scale bar: 100 nm. **(C)** Western blot analysis of the EV markers CD9 and CD63, and TOM20 (n = 3). **(D)** Nanoparticle tracking analysis of EV. **(E)** iWAT weight quantification (n = 6). **(F**–**G)** Quantification of Con-EV, Cold-3dEV, and Cold-7dEV particles and protein from 100 mg iWAT (n = 6). **(H**–**J)** Quantification of Western blotting bands of EV markers CD9, CD63 and TOM20, related to (**C**) (n = 3). *P < 0.05, **P < 0.01, ***P < 0.001.

EV derived from adipose tissue are important mediators of its biological functions, and cold stimulation has been reported to alter their composition and function [37]. Hence, we next sought to determine whether STIMCE-induced metabolic reprogramming of iWAT influenced the biogenesis and composition of the secreted EV. Following the experimental workflow depicted in Fig. 2A, EV were successfully isolated from the iWAT of Con, Cold-3d, and Cold-7d mice via a sequential differential ultracentrifugation procedure and are hereafter referred to as Con-EV, Cold-3dEV, and Cold-7dEV, respectively. Morphological characterization via transmission electron microscopy (TEM) revealed that the isolated EV particles in all groups exhibited the typical cup-shaped, bilayered vesicular structure (Fig. 2B). Western blot analysis showed that all EV populations contained the classic EV markers CD9 and CD63. In particular, the expression levels of these markers were significantly higher in Cold-3dEV and Cold-7dEV than in Con-EV (Fig. 2C–H–I). To quantify the yield of EV secretion, we performed nanoparticle tracking analysis (NTA) and protein quantification. Strikingly, despite the comparable iWAT mass across all groups (Fig. 2E), both the total number of EV particles and the protein yield per gram of tissue were progressively and significantly increased following STIMCE (Fig. 2D–F–G). Interestingly, western blotting revealed an obvious increase in TOM20 expression in the STIMCE-EV, with the highest expression observed in the Cold-7dEV group (Fig. 2C–J). This suggests that STIMCE induced the selective packaging of mitochondrial components into EV. Taken together, these findings demonstrate that STIMCE not only promotes the browning of iWAT but also increases EV production and the enrichment of mitochondrial components.

### 3.3 Fabrication and characterization of AAM for EV loading (AAM@STIMCE-EV) and sustained release

**Fig. 3:**
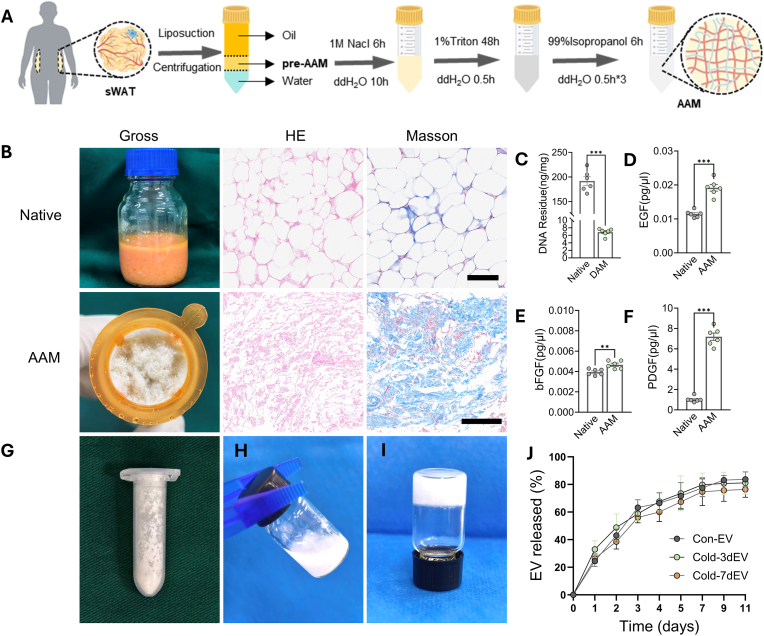
Fabrication and characterization of AAM for EV loading (AAM@STIMCE-EV) and sustained release. **(A)** Schematic illustration of subcutaneous white adipose tissue (sWAT) isolation and AAM preparation. **(B)** Gross morphology, H&E, and Masson's trichrome staining of native sWAT and AAM. Scale bar: 100 μm. **(C)** Quantification of residual DNA in AAM and native sWAT (n = 6). **(D**–**F)** Contents of the growth factors bFGF, PDGF, and EGF in AAM and native sWAT (n = 6). **(G)** AAM powder. **(H**–**I)** AAM hydrogel at room temperature and 37 °C, respectively. **(J)** Controlled release profiles of the AAM@Con-EV, AAM@Cold-3dEV, and AAM@Cold-7dEV hydrogels (n = 6). *P < 0.05, **P < 0.01, ***P < 0.001.

To develop a biocompatible scaffold capable of delivering EV in vivo, we prepared AAM from subcutaneous liposuction samples using a stepwise decellularization procedure (Fig. 3A). The processed AAM appeared as a white, translucent, sponge-like material, morphologically distinct from native adipose tissue. Histological evaluation via H&E and Masson's trichrome staining confirmed the complete removal of cellular components while preserving a highly porous, collagen-rich extracellular architecture (Fig. 3B). Quantitative DNA assays demonstrated minimal residual DNA content (<50 ng/mg tissue), meeting commonly accepted criteria for decellularized tissues and suggesting efficient decellularization (Fig. 3C). Notably, compared with native adipose tissue, the AAM exhibited elevated concentrations of key growth factors, including EGF, bFGF, and PDGF (Fig. 3D–F). The lyophilized AAM was then ground into powder (Fig. 3G). After being digested with pepsin and neutralized, the AAM hydrogel remained liquid at room temperature and spontaneously solidified at 37 °C, demonstrating its thermosensitive properties (Fig. 3H and I). Then, the AAM hydrogel was mixed with EV solutions to prepare the AAM@EV composite hydrogel. An in vitro release assay showed a desirable biphasic release with an initial burst within the first 72 h followed by a prolonged release over 11 days, with no significant differences observed among the AAM@Con-EV, AAM@Cold-3dEV, and AAM@Cold-7dEV groups (Fig. 3J). Together, these findings suggest that the fabricated human AAM hydrogel scaffolds were suitable for EV loading and controlled release.

### 3.4 AAM@STIMCE-EV promotes endothelial angiogenic functions in vitro

**Fig. 4:**
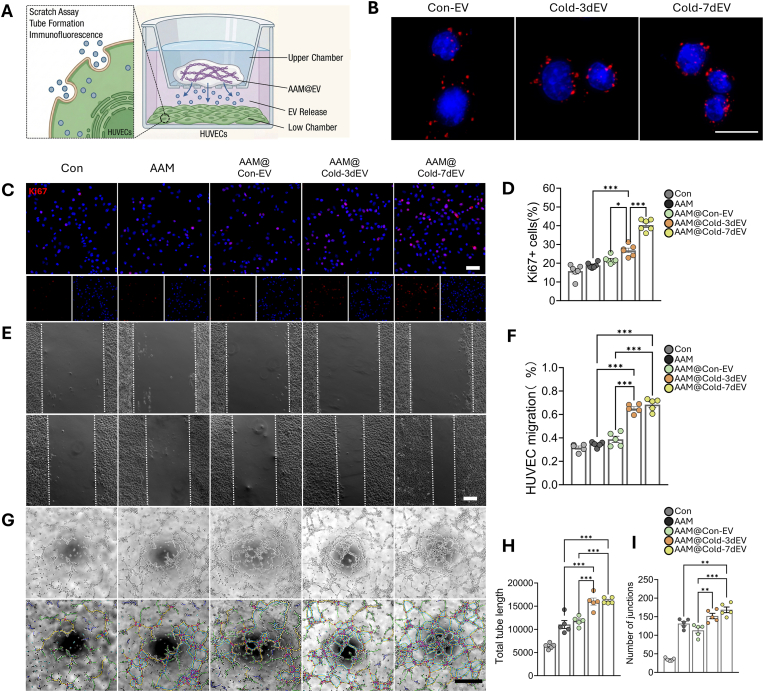
AAM@STIMCE-EV promotes endothelial angiogenic functions in vitro. **(A)** Schematic illustration of HUVECs cocultured with AAM@EV composite scaffolds in a Transwell system. **(B)** Representative fluorescence images showing the cellular uptake of DiI-labeled Con-EV, Cold-3dEV, and Cold-7dEV by HUVECs. **(C**–**D)** Ki67 staining and quantification of HUVECs after 48 h of coculture with different AAM@EV scaffolds (n = 6). Scale bar: 100 μm. **(E**–**F)** Scratch wound healing assays and quantification of migration area after 24 h of coculture (n = 5). Scale bar: 200 μm. **(G**–**I)** Tube formation assay and corresponding quantification of total tube length and number of junctions after 48 h of coculture (n = 5). Scale bar: 200 μm. *P < 0.05, **P < 0.01, ***P < 0.001.

It is well established that adequate vascularization is essential for the successful regeneration of engineered adipose tissue [4]. Therefore, we further explored the effects of AAM scaffolds loaded with different EV populations on the angiogenic capacity of HUVECs. We established an in vitro coculture system utilizing the AAM@EV composite scaffolds and HUVECs in a Transwell arrangement, as depicted in Fig. 4A. To initially determine whether these EV were effectively internalized, we incubated DiI-labeled EV with HUVECs for 24 h. Subsequent fluorescence imaging revealed a robust red signal within HUVECs across all groups, indicating that the AAM-released EV were efficiently internalized by HUVECs (Fig. 4B). We then tested the biological effect of AAM@EV on HUVECs proliferation. At 48 h after coculture, the Ki67-positive proportion was much higher in HUVECs treated with all AAM@EV groups than in the AAM alone group or the untreated control. Notably, the AAM@Cold-7dEV group exhibited the strongest Ki67 expression (Fig. 4C and D). A scratch assay was carried out to evaluate the migration ability of HUVECs. Compared with the AAM group and AAM@Con-EV group, AAM@Cold-3dEV and AAM@Cold-7dEV treatment significantly accelerated HUVEC migration (Fig. 4E and F). The tube formation assay showed that HUVECs in the AAM@Cold-3dEV and AAM@Cold-7dEV groups displayed significantly elevated total tube lengths and a higher number of junctions (Fig. 4G–I). Taken together, these data indicate that AAM@STIMCE-EV successfully promotes the proliferation, migration and tube formation of HUVECs, which are essential to support adipose tissue regeneration.

### 3.5 AAM@STIMCE-EV drives metabolic reprogramming and mitochondrial biogenesis in hSVF cells

**Fig. 5:**
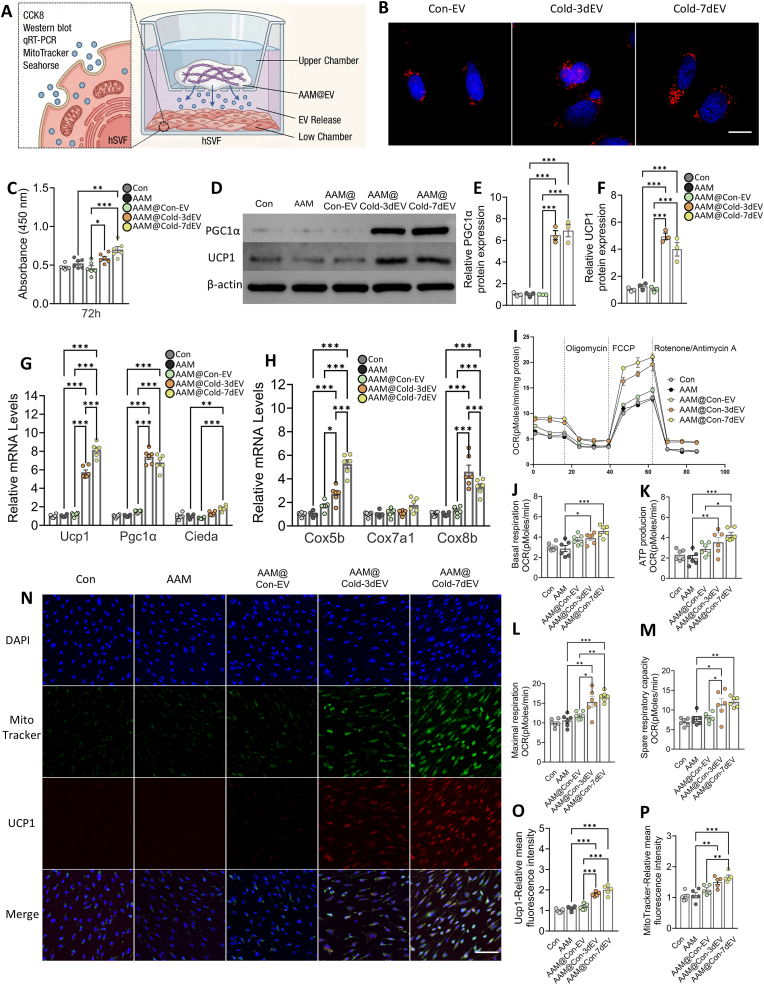
AAM@STIMCE-EV drives metabolic reprogramming and mitochondrial biogenesis in hSVF cells. (**A**) Schematic illustration of hSVF cells cocultured with AAM@EV composite scaffolds in a Transwell system. (**B**) Representative fluorescence images showing the cellular uptake of DiI-labeled Con-EV, Cold-3dEV, and Cold-7dEV by hSVF cells. (**C**) CCK-8 assay of hSVF cells after 72 h of coculture with different AAM@EV scaffolds (n = 6). (**D–F**) Western blot analysis of PGC-1α and UCP1 protein levels in hSVF cells after 48 h of coculture with different AAM@EV scaffolds (n = 3). (**G-H**) Relative mRNA expression of thermogenic and mitochondrial-related genes in hSVF cells (n = 6). (**I**) Representative traces of the oxygen consumption rate (OCR) in hSVF cells treated with different AAM@EV scaffolds for 48 h (n = 6). (**J–M**) Quantification of basal respiration, ATP production, maximal respiration, and spare respiratory capacity (n = 6). (**N**) Representative fluorescence images showing MitoTracker (green) and UCP1 (red) expression in hSVF cells treated with different AAM@EV scaffolds for 48 h (n = 5). Scale bar: 100 μm. (**O–P**) Quantification of mean fluorescence intensity for MitoTracker and UCP1 expression in (N) (n = 5). *P < 0.05, **P < 0.01, ***P < 0.001. (For interpretation of the references to colour in this figure legend, the reader is referred to the Web version of this article.)

Given that successful adipose tissue regeneration relies heavily on the activation and metabolic adaptation of progenitor cells [14,17], we next investigated the regulatory effects of AAM@STIMCE-EV on primary hSVF cells. hSVF cells were successfully isolated from human lipoaspirate, and their multipotency was confirmed via adipogenic and osteogenic differentiation assays (Supplementary Fig. 1). hSVF cells were cocultured with AAM@EV composite scaffolds, as illustrated in Fig. 5A. Consistent with our findings in endothelial cells, fluorescence microscopy revealed that DiI-labeled EV were effectively internalized by hSVF cells within 24 h, demonstrating the successful delivery of EV cargo to hSVF cells (Fig. 5B). CCK-8 assays demonstrated that hSVF cells treated with AAM@Cold-3dEV or AAM@Cold-7dEV exhibited significantly higher proliferation rates compared with those in the AAM alone and AAM@Con-EV groups after 72 h of coculture (Fig. 5C). Beyond proliferation, we sought to determine whether STIMCE-EV could trigger a thermogenic shift in hSVF cells. Western blotting analysis revealed a marked upregulation of the key thermogenic proteins Ucp1 and Pgc1α in hSVF cells following treatment with AAM@Cold-3dEV or AAM@Cold-7dEV (Fig. 5D–F). Consistently, qRT-PCR analysis showed marked upregulation of thermogenic genes (Ucp1, Pgc1α, and Cidea) and mitochondrial oxidative phosphorylation genes (Cox5b, Cox7a1, and Cox8b) (Fig. 5G and H). Seahorse analysis then revealed that basal respiration, ATP production, maximal respiration, and spare respiratory capacity were progressively enhanced in the AAM@Cold-3dEV and AAM@Cold-7dEV groups. The values reached their peak in the AAM@Cold-7dEV group (Fig. 5I–M). MitoTracker and UCP1 staining further validated this functional metabolic activation. The AAM@STIMCE-EV group displayed a denser and more interconnected mitochondrial network. These groups also showed robust Ucp1 expression (Fig. 5N–P). Collectively, these findings reveal that AAM@STIMCE-EV can effectively reprogram the metabolic phenotype of hSVF cells, stimulating mitochondrial biogenesis and a thermogenic phenotype that may be essential for functional adipose tissue regeneration.

### 3.6 AAM@STIMCE-EV enhances in vivo adipose tissue regeneration

**Fig. 6:**
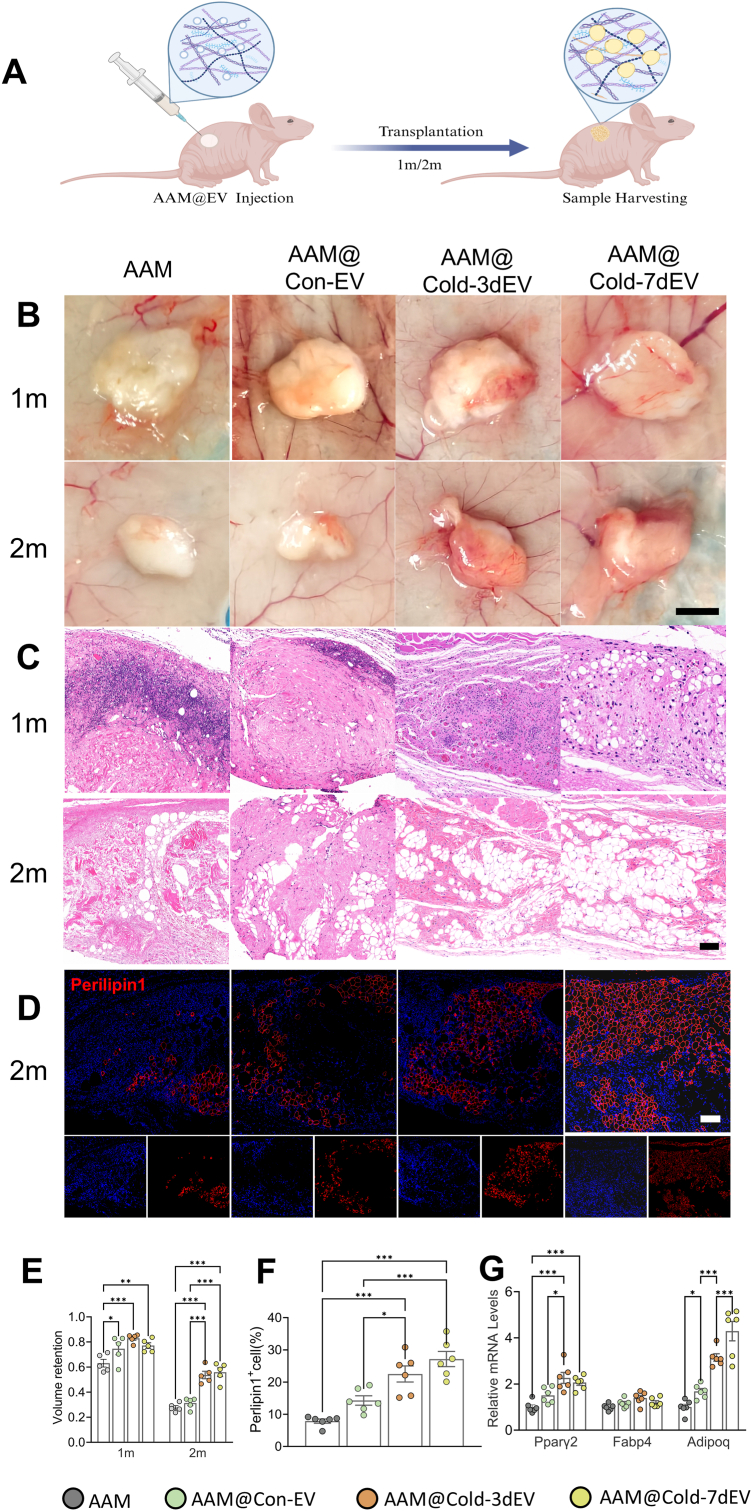
AAM@STIMCE-EV enhances in vivo adipose tissue regeneration. (**A**) Schematic illustration of the in vivo transplantation of different AAM@EV hydrogel scaffolds. Created in BioRender. Zhu, S. (2026) https://BioRender.com/5d8c8rl. Grafts were harvested and analyzed at 1 and 2 months post-transplantation. (**B, E**) Macroscopic views of grafts at 1 and 2 months and corresponding quantification of volume retention (n = 5). Scale bar: 0.3 cm. (**C**) Representative H&E staining images of different AAM@EV grafts. Scale bar: 100 μm. (**D, F**) Perilipin1 immunofluorescence and quantification of the percentage of Perilipin1^+^ cells at 2 months post-transplantation (n = 6). Scale bar: 200 μm. (**G**) Relative mRNA expression of adipogenesis-related genes in different AAM@EV grafts at 2 months post-transplantation (n = 6). *P < 0.05, **P < 0.01, ***P < 0.001.

To evaluate the in vivo regenerative potential of AAM@STIMCE-EV, we subcutaneously injected AAM hydrogel alone, AAM@Con-EV, AAM@Cold-3dEV, or AAM@Cold-7dEV hydrogel into BALB/c nude mice. Grafts were harvested at 1 and 2 months post-implantation for comprehensive analysis (Fig. 6A). Morphological assessment revealed that all AAM@EV groups maintained significantly greater volume retention compared with the AAM alone group at the 1-month time point (Fig. 6B–E). Grafts in the AAM@Cold-3dEV and AAM@Cold-7dEV groups showed substantially greater volume retention at 2 months compared with both the AAM and AAM@Con-EV groups (Fig. 6B–E). Consistent with gross observations, H&E staining revealed early adipose regeneration in AAM@Cold-7dEV implants as soon as 1 month. This progressed to abundant newly formed adipocytes by 2 months (Fig. 6C). Conversely, the control AAM grafts were mainly composed of fibrotic tissue with limited adipocyte formation. Although AAM@Con-EV treatment improved the regenerative outcomes, the overall effect was limited compared with the AAM@Cold-3dEV and AAM@Cold-7dEV groups (Fig. 6C). Immunofluorescence staining demonstrated a significantly higher proportion of Perilipin1^+^ adipocytes in AAM@Cold-3dEV and AAM@Cold-7dEV grafts at 2 months. The AAM@Cold-7dEV group displayed the highest Perilipin1^+^ proportion (Fig. 6D–F). qRT-PCR further validated these histological findings. AAM@STIMCE-EV grafts showed elevated mRNA levels of adipogenesis-associated genes (Pparg, Fabp4, and Adipoq) by 2 months (Fig. 6G). Finally, we confirmed the systemic safety of the AAM@STIMCE-EV hydrogel. Blood routine, blood biochemistry, and organ staining showed no obvious toxicity in the treated mice (Supplementary Fig. 2). Collectively, these findings suggest that AAM loaded with STIMCE-EV promotes in vivo adipose tissue regeneration by enhancing graft retention, adipogenic commitment, and structural remodeling.

### 3.7 STIMCE-EV orchestrate metabolic reprogramming of AAM grafts

**Fig. 7:**
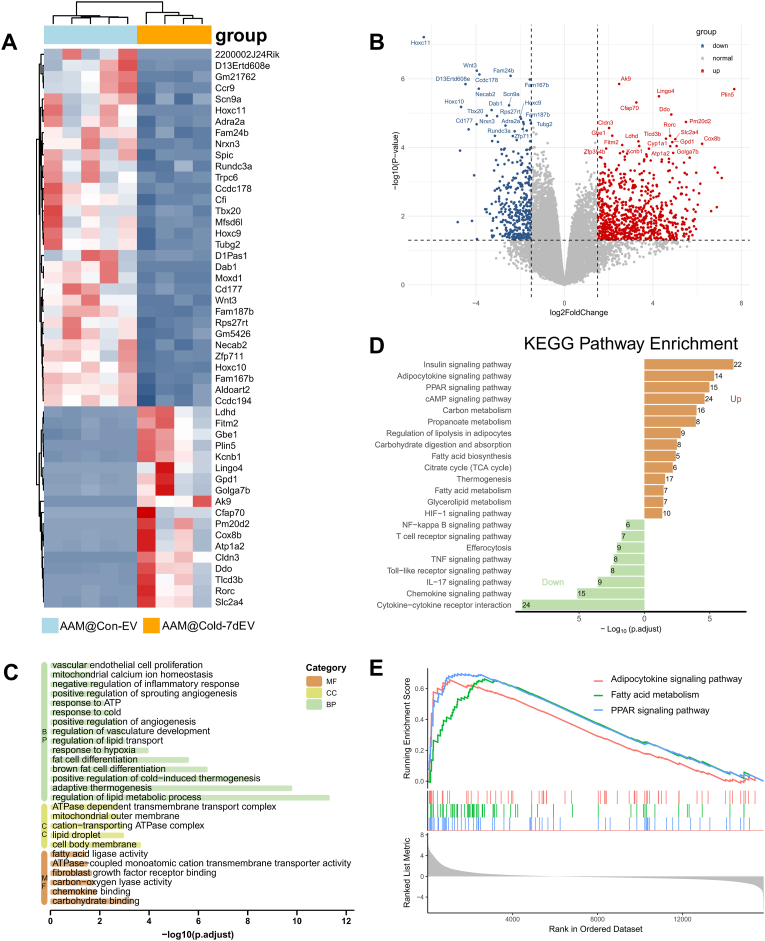
STIMCE-EV orchestrate metabolic reprogramming of AAM grafts. Transcriptomic profiling was performed on AAM grafts harvested 2 months post-implantation from the AAM@Con-EV (n = 5) and AAM@Cold-7dEV groups (n = 4). (**A, B**) Hierarchical clustering heatmap and volcano plot displaying the global transcriptional profiles and DEGs. (**C, D**) GO and KEGG enrichment analyses identifying the top-regulated biological processes and signaling pathways associated with the DEGs. (**E**) GSEA plots revealing the positive enrichment of metabolism-related pathways (Adipocytokine signaling pathway, Fatty acid metabolism, and PPAR signaling pathway) in the AAM@Cold-7dEV grafts.

To elucidate the mechanism by which AAM@STIMCE-EV grafts enhance adipose tissue regeneration, transcriptome sequencing was performed to compare the AAM@Cold-7dEV and AAM@Con-EV grafts. As shown in the volcano plot and heatmap, the transcriptional profiles exhibited marked differences (Fig. 7A and B). Subsequent Gene Ontology (GO) enrichment analysis associated the identified differentially expressed genes (DEGs) primarily with the regulation of lipid metabolic processes, as well as critical thermogenic pathways, specifically adaptive and cold-induced thermogenesis. Other enriched processes included fat cell differentiation, lipid transport, and responses to hypoxia and angiogenesis (Fig. 7C). At the cellular component level, DEGs were associated with the cell body membrane, lipid droplets, cation-transporting ATPase complex, and the mitochondrial outer membrane. Molecular function analysis identified terms such as carbohydrate binding, chemokine binding, carbon–oxygen lyase activity and FGF receptor binding (Fig. 7C). Kyoto Encyclopedia of Genes and Genomes (KEGG) analysis also highlighted the enrichment of thermogenic and metabolic pathways including insulin signaling, adipocytokine signaling, PPAR signaling, cAMP signaling, and carbon metabolism pathways (Fig. 7D). Fatty acid biosynthesis, the TCA cycle, and glycerolipid metabolism were also strongly enriched in upregulated pathways. Conversely, inflammatory and immune-related pathways were enriched in the downregulated gene sets, including cytokine–cytokine receptor interaction, chemokine signaling, TNF-α, IL-17, and NF-κB signaling (Fig. 7D). Consistent with the KEGG results, gene set enrichment analysis (GSEA) demonstrated positive enrichment of adipocytokine, fatty acid metabolism, and PPAR signaling pathways (Fig. 7E). Collectively, this transcriptomic landscape reveals that STIMCE-EV orchestrate a marked metabolic reprogramming of the grafts. This process fosters a metabolically active microenvironment that supports functional adipose regeneration.

### 3.8 STIMCE-EV promote immunomodulation and neovascularization of AAM grafts

**Fig. 8:**
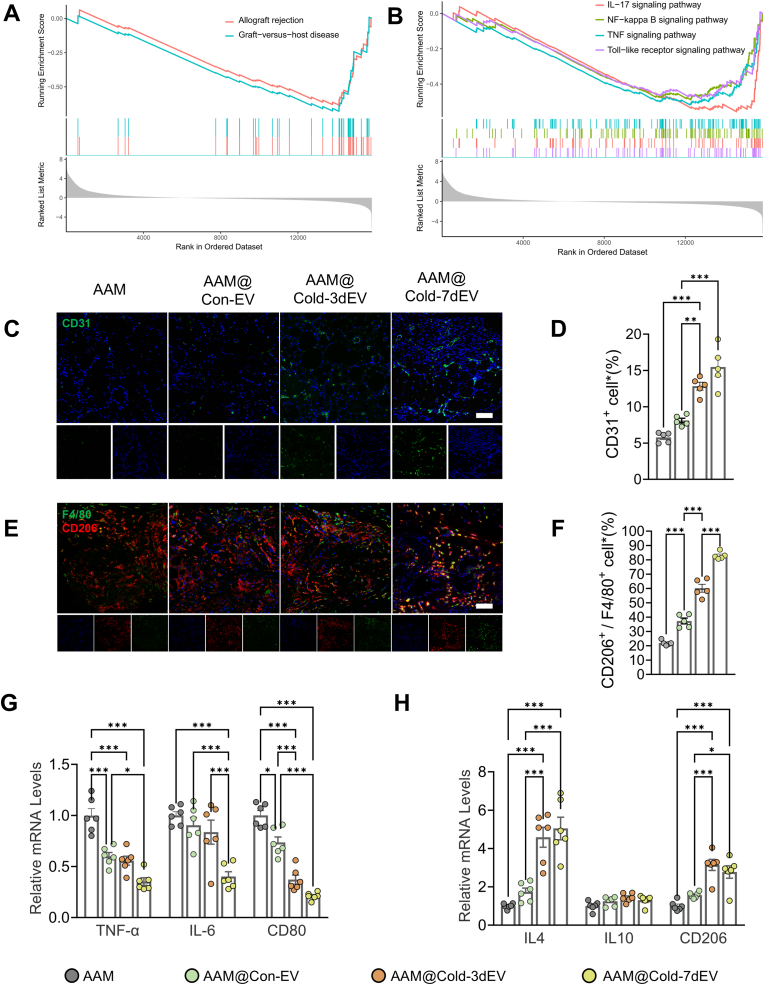
STIMCE-EV promote immunomodulation and neovascularization of AAM grafts. (**A–B**) GSEA plots revealing the negative enrichment of pathways associated with immune rejection (Allograft rejection and Graft-versus-host disease) and inflammatory signaling (IL-17, NF-kappa B, TNF, and Toll-like receptor signaling pathway) in the AAM@Cold-7dEV group. (**C–D**) Representative immunofluorescence staining and quantification of the CD31^+^ cells at 2 months post-transplantation (n = 5). Scale bar: 100 μm. (**E–F**) Immunofluorescence staining and quantification of macrophage polarization. F4/80^+^ (green) and CD206^+^ (red) were used to identify the M2-like macrophage population within different grafts (n = 5). Scale bar: 100 μm. (**G–H**) Relative mRNA expression of pro-inflammatory markers (TNF-α, IL-6, and CD80) and anti-inflammatory markers (IL-4, IL-10, and CD206) in different AAM@EV grafts at 2 months post-transplantation (n = 6). *P < 0.05, **P < 0.01, ***P < 0.001. (For interpretation of the references to colour in this figure legend, the reader is referred to the Web version of this article.)

The successful regeneration of AAM scaffolds is critically dependent on a favorable host immune response and rapid vascularization. Although decellularization significantly minimizes inherent immunogenicity, the implanted AAM remains capable of eliciting a host immune response that may determine the eventual regenerative outcome [17,26]. To assess this, we first analyzed the transcriptomic profile of the grafts. GSEA revealed a significant negative enrichment of pathways associated with Allograft rejection and Graft-versus-host disease in the AAM@Cold-7dEV group, suggesting a potentially attenuated immune rejection profile. Indeed, several key inflammatory signaling pathways (IL-17, NF-κB, TNF-α, and Toll-like receptor) were found to be significantly downregulated in AAM@Cold-7dEV grafts compared with AAM@Con-EV grafts (Fig. 8A and B). Neovascularization was then evaluated by CD31 immunofluorescence staining (Fig. 8C). Although AAM@Con-EV grafts showed better vascularization than AAM alone, stronger angiogenic responses were observed in both AAM@Cold-3dEV and AAM@Cold-7dEV grafts (Fig. 8D). Co-staining for F4/80 and CD206 showed a gradual increase in CD206^+^F4/80^+^ cells across the four groups, from AAM alone to AAM@Con-EV, AAM@Cold-3dEV, and AAM@Cold-7dEV (Fig. 8E and F). These results support a transition toward a pro-regenerative M2 phenotype associated with STIMCE-EV treatment. Concurrently, qRT-PCR analysis further confirmed the elevated expression of anti-inflammatory genes (IL4, IL10, and CD206) and the reduction of pro-inflammatory markers (TNF-α, IL-6, and CD80) in the STIMCE-EV-treated grafts (Fig. 8G and H). Taken together, these findings reveal that while Con-EV confer modest immunomodulatory and pro-angiogenic support to the AAM scaffold, STIMCE-EV significantly enhance these effects. Hence, AAM@STIMCE-EV may establish a pro-angiogenic and anti-inflammatory microenvironment that is essential for functional adipose tissue regeneration.

### 3.9 AAM@STIMCE-EV grafts regenerate metabolically active adipose tissue and enhance systemic thermoregulation in host mice under cold stress

**Fig. 9:**
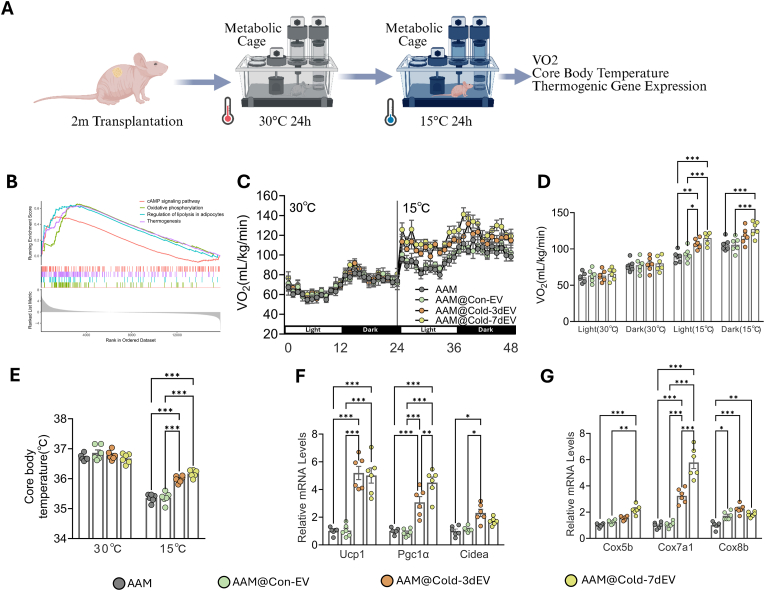
AAM@STIMCE-EV grafts regenerate metabolically active adipose tissue and enhance systemic thermoregulation in host mice under cold stress. **(A)** Schematic illustration of the metabolic cage assessment. Created in BioRender. Zhu, S. (2026) https://BioRender.com/5d8c8rl. At 2 months post-transplantation, host mice were sequentially exposed to thermoneutrality (30 °C) and cold stress (15 °C) for 24 h each to monitor metabolic parameters. **(B)** GSEA plots revealing the positive enrichment of thermogenesis-related pathways (cAMP signaling pathway, Oxidative phosphorylation, Regulation of lipolysis in adipocytes, and Thermogenesis) in the AAM@Cold-7dEV group. **(C**–**D)** Whole-body oxygen consumption profiles of host mice at 30 °C and 15 °C (n = 6). **(E)** Core body temperature of host mice at 30 °C and 15 °C (n = 6). **(F**–**G)** Relative mRNA expression of thermogenesis-related genes (Ucp1, Pgc1a, and Cidea) and mitochondrial oxidative phosphorylation-related genes (Cox5b, Cox7a1, and Cox8b) in AAM grafts after 15 °C cold exposure (n = 6). *P < 0.05, **P < 0.01, ***P < 0.001.

A critical hallmark of functional adipose tissue regeneration is the restoration of key metabolic capabilities, particularly the capacity for adaptive thermogenesis, alongside structural reconstitution. We therefore sought to determine whether these newly formed adipose depots were metabolically functional and could contribute to systemic energy homeostasis. As illustrated in Fig. 9A, at 2 months post-transplantation, host nude mice were sequentially exposed to thermoneutrality (30 °C) and cold stress (15 °C) for 24 h each to monitor metabolic parameters. GSEA analysis revealed a significant enrichment of pathways including cAMP signaling, oxidative phosphorylation, and the regulation of lipolysis (Fig. 9B). In parallel with these molecular signatures, indirect calorimetry showed that although O_2_ consumption was comparable among all groups at 30 °C, mice engrafted with AAM@Cold-3dEV and AAM@Cold-7dEV showed significant increases in O_2_ consumption at 15 °C during both light and dark phases (Fig. 9C and D). We then tested whether the AAM@STIMCE-EV grafts influenced the cold tolerance of host mice systemically (Fig. 9E). While the core body temperature of all groups was similar and stable at 30 °C, when challenged with 15 °C for 24 h, the mice bearing AAM alone or AAM@Con-EV grafts showed a sharp drop in body temperature. However, this cold response was significantly rescued by grafting AAM@Cold-3dEV and AAM@Cold-7dEV into mice, as these mice displayed significantly greater core body temperatures (Fig. 9E). Consistent with this metabolic finding, qRT-PCR analysis confirmed an elevated expression of major thermogenic genes (Ucp1, Pgc1α, and Cidea), as well as mitochondrial oxidative genes (Cox5b, Cox7a1, and Cox8b) (Fig. 9F and G). To determine whether AAM implantation itself contributed to systemic metabolic changes, we also compared non-implanted Con mice with AAM-implanted mice. AAM implantation alone did not significantly alter whole-body O_2_ consumption or core body temperature under either 30 °C or 15 °C conditions (Supplementary Fig. 3A–C). Hence, the improved systemic thermoregulation observed in the AAM@STIMCE-EV group may not be attributable to the intrinsic effect of AAM implantation alone. In summary, these data show that AAM@STIMCE-EV implants regenerate structurally and functionally thermogenic adipose tissue. This regenerated tissue actively participates in adaptive thermogenesis in response to cold exposure, highlighting its therapeutic potential for metabolic disorders.

### 3.10 mmu-miR-296-3p identified as a potential key mediator of STIMCE-EV

**Fig. 10:**
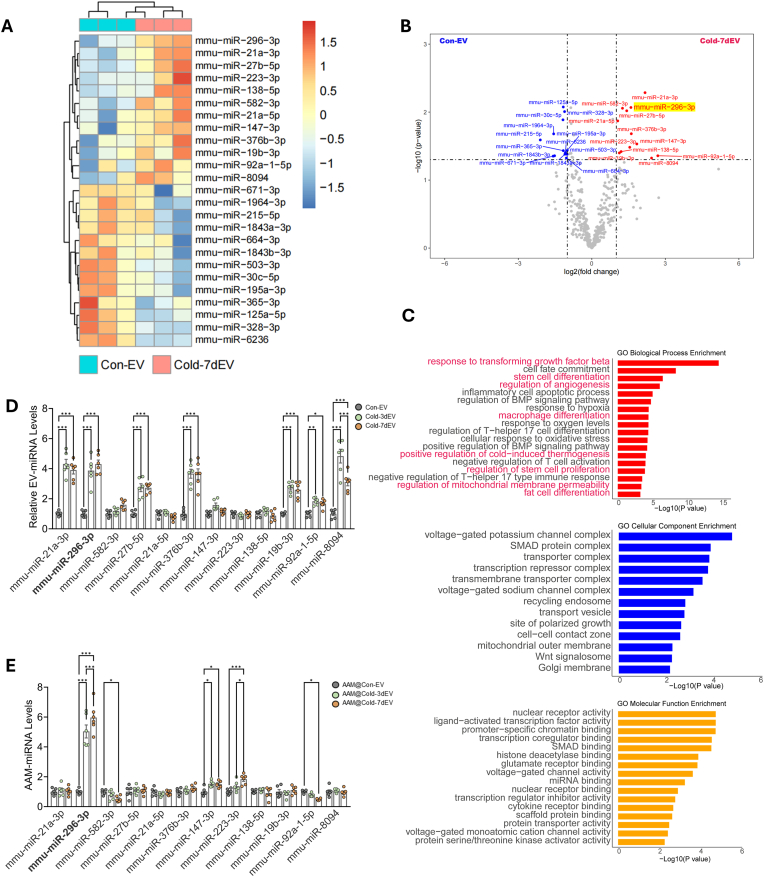
mmu-miR-296-3p identified as a potential key mediator of STIMCE-EV. **(A)** Hierarchical clustering heatmap of miRNA expression profiles in Cold-7dEV and Con-EV samples (n = 3). Differentially expressed miRNAs were identified based on the criteria of P < 0.05 and |log2 FC| ≥ 1. **(B)** Volcano plot highlighting the significantly upregulated and downregulated miRNAs. **(C)** GO enrichment analysis of the predicted target genes of the identified DEMs. **(D)** qRT-PCR of candidate upregulated miRNAs in Con-EV, Cold-3dEV and Cold-7dEV (n = 6). **(E)** Expression levels of upregulated miRNAs in AAM@Con-EV, AAM@Cold-3dEV and AAM@Cold-7dEV grafts at 2 months post-transplantation (n = 6). *P < 0.05, **P < 0.01, ***P < 0.001.

To elucidate the molecular mediators governing STIMCE-EV-induced AAM regeneration, we performed miRNA sequencing on the EV cargo from Cold-7dEV and Con-EV. Differential expression analysis identified 12 upregulated and 13 downregulated miRNAs in the Cold-7dEV group using a threshold of |log_2_FC| > 1 and P < 0.05 (Fig. 10A and B). Subsequent GO enrichment analysis of the predicted target genes for these differentially expressed miRNAs revealed significant associations with critical metabolic processes, including fat cell differentiation, angiogenesis, thermogenesis, and immune modulation (Fig. 10C). Notably, these functional categories were highly consistent with the transcriptomic enrichment patterns previously observed in the regenerated grafts (Fig. 7). qRT-PCR was performed to validate the sequencing findings for the upregulated candidate miRNAs. We found that mmu-miR-21a-3p, mmu-miR-296-3p, mmu-miR-27b-5p, mmu-miR-376-3p, mmu-miR-19b-3p, mmu-miR-92a-1-5p and mmu-miR-8094 were highly expressed in Cold-7dEV compared with Con-EV (Fig. 10D). Notably, mmu-miR-296-3p exhibited the most pronounced upregulation in Cold-7dEV. Crucially, its elevated expression was not only confirmed within the isolated EV but was also found to be persistently high within AAM@Cold-7dEV grafts at 2 months post-transplantation (Fig. 10E). This finding distinguished mmu-miR-296-3p from other candidate miRNAs, which were enriched in Cold-7dEV but did not show the same sustained elevation in AAM@Cold-7dEV grafts at 2 months, suggesting that their effects may be more transient or less directly associated with long-term graft remodeling. Based on these findings, we hypothesized that mmu-miR-296-3p serves as a key molecular mediator driving the STIMCE-EV-induced metabolic activation and functional regeneration of the AAM scaffold.

### 3.11 mmu-miR-296-3p drives hSVF cells metabolic reprogramming and mediates the adipose regeneration of AAM@STIMCE-EV in vivo

**Fig. 11:**
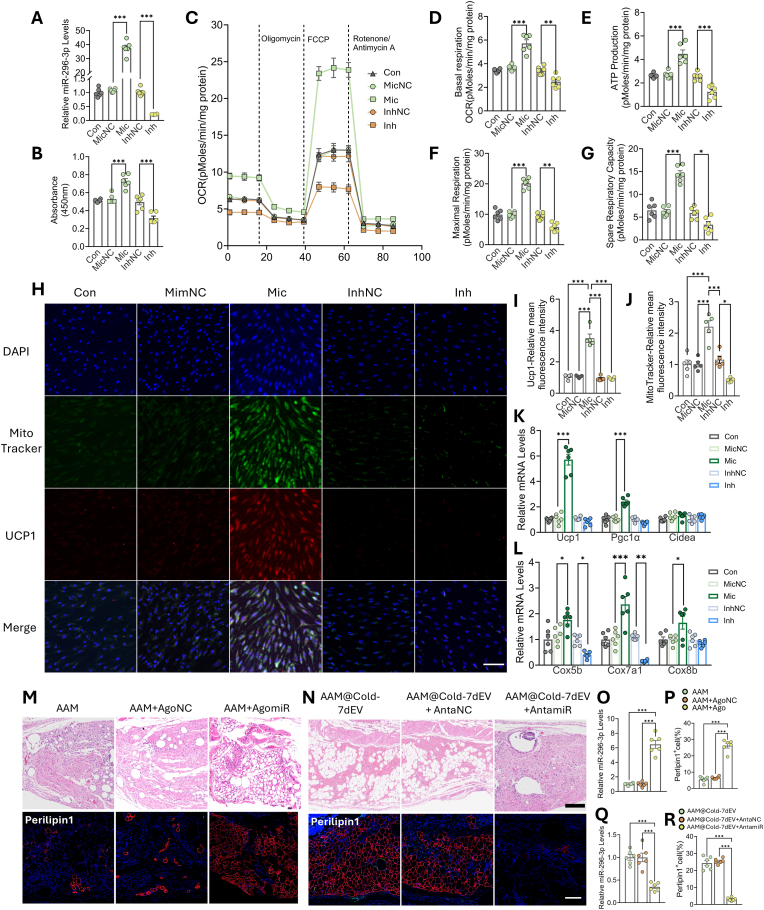
mmu-miR-296-3p drives hSVF cells metabolic reprogramming and mediates the adipose regeneration of AAM@STIMCE-EV in vivo. **(A)** Validation of mmu-miR-296-3p expression in hSVF cells alone (Con) or treated with mmu-miR-296-3p mimic (Mic), inhibitor (Inh), or their respective negative controls (MicNC, InhNC) (n = 6). **(B)** CCK-8 assay measuring hSVF cells proliferation after 48 h of treatment (n = 6). **(C)** OCR profiles of hSVF cells after 48 h of treatment (n = 6). **(D**–**G)** Quantification of basal respiration, ATP production, maximal respiration, and spare respiratory capacity (n = 6). **(H)** Representative immunofluorescence images of MitoTracker (green) and UCP1 (red) in hSVF cells after 48 h of treatment (n = 5). Scale bar: 100 μm. **(I**–**J)** Quantification of mean fluorescence intensity for MitoTracker and UCP1 in (H). **(K**–**L)** Relative mRNA expression of thermogenesis-related genes (Ucp1, Pgc1a, and Cidea) and mitochondrial oxidative phosphorylation-related genes (Cox5b, Cox7a1, and Cox8b) in hSVF cells after 48 h of treatment (n = 6). **(M)** Representative H&E and Perilipin1 immunofluorescence images of AAM hydrogels mixed with mmu-miR-296-3p AgomiR or AgoNC to assess whether mmu-miR-296-3p supplementation promotes adipose regeneration. **(N)** Representative H&E and Perilipin1 immunofluorescence images of AAM@Cold-7dEV hydrogels mixed with mmu-miR-296-3p AntamiR or AntaNC to determine whether the pro-adipogenic effect could be reversed. Scale bar: 200 μm. **(O)** Relative mmu-miR-296-3p expression in (M). **(P)** Quantification of the percentage of Perilipin1^+^ cells in (M). **(Q)** Relative mmu-miR-296-3p expression in (N). **(R)** Quantification of the percentage of Perilipin1^+^ cells in (N). *P < 0.05, **P < 0.01, ***P < 0.001. (For interpretation of the references to colour in this figure legend, the reader is referred to the Web version of this article.)

To elucidate the functional role of mmu-miR-296-3p, hSVF cells were transfected with chemically synthesized miRNA mimics (Mic), inhibitors (Inh), and their respective negative controls (MicNC, InhNC). qRT-PCR analysis confirmed that Mic and Inh treatments effectively increased and suppressed mmu-miR-296-3p expression in hSVF cells, respectively (Fig. 11A). CCK-8 assays demonstrated that Mic treatment significantly enhanced hSVF cells proliferation, whereas Inh treatment markedly reduced cell growth (Fig. 11B). Seahorse analysis was performed to determine whether mmu-miR-296-3p could metabolically reprogram hSVF cells (Fig. 11C). Indeed, Mic-treated hSVF cells exhibited a significant increase in key oxygen consumption rate parameters, including basal respiration, ATP production, maximal respiration, and spare respiratory capacity. Conversely, Inh treatment produced the opposite effect (Fig. 11C–G). MitoTracker and Ucp1 immunofluorescence staining further corroborated this functional metabolic activation (Fig. 11H–J). The Mic-treated hSVF cells displayed a significantly higher mean fluorescence intensity for both MitoTracker and Ucp1 expression. In contrast, mmu-miR-296-3p Inh treatment significantly inhibited the mean fluorescence intensity of MitoTracker, although the mean fluorescence intensity of Ucp1 was not significantly altered compared with the control group (Fig. 11H–J). Consistently, qRT-PCR analysis confirmed that mmu-miR-296-3p Mic treatment elevated the expression of thermogenic genes (Ucp1, Pgc1a, and Cidea) and oxidative phosphorylation genes (Cox5b, Cox7a1, and Cox8b), while the Inh treatment suppressed their expression (Fig. 11K and L). No significant differences were observed in the MicNC or InhNC groups, reinforcing the specificity of these metabolic changes.

Finally, to validate whether exogenous supplementation of mmu-miR-296-3p could promote AAM adipogenesis in vivo, we co-administered a mmu-miR-296-3p AgomiR with AAM hydrogels (Fig. 11M). The AgomiR effectively elevated mmu-miR-296-3p levels in the grafts at 2 months post-implantation, confirming the efficacy of the delivery system (Fig. 11O). The proportion of Perilipin1^+^ cells in the AAM+AgomiR group was significantly higher than that in the AAM alone and AAM + AgoNC groups (Fig. 11P), indicating that the AgomiR significantly promoted AAM adipose regeneration at 2 months post-transplantation. Furthermore, we investigated whether exogenous inhibition of mmu-miR-296-3p could reverse the enhanced adipogenesis induced by AAM@Cold-7dEV (Fig. 11N). The AntamiR successfully suppressed mmu-miR-296-3p expression within the grafts (Fig. 11Q). The proportion of Perilipin1^+^ cells in the AAM@Cold-7dEV + AntamiR group was significantly lower than that in the AAM@Cold-7dEV + AntaNC group (Fig. 11R). These findings demonstrate that the AntamiR treatment significantly reversed the STIMCE-EV-induced adipogenesis, identifying mmu-miR-296-3p as a critical mediator of AAM regeneration. Collectively, these results demonstrate that mmu-miR-296-3p is a critical functional cargo of STIMCE-EV, mediating hSVF cells metabolic reprogramming and driving the in vivo adipose regeneration of the AAM@STIMCE-EV scaffold.

To further identify the downstream target involved in metabolic regulation by mmu-miR-296-3p, candidate target genes were predicted using TargetScan and miRDB. The overlapping genes were then screened according to their relevance to mitochondrial stress regulation. Among these candidates, SOCS6, a member of the suppressor of cytokine signaling family, was selected for further validation because it was predicted by both databases and has been linked to mitochondrial stress-related processes in previous studies [45,46] (Supplementary Fig. 4A). TargetScan analysis identified a conserved miR-296-3p binding site within the human SOCS6 3′-UTR. Therefore, wild-type and mutant SOCS6 3′-UTR luciferase reporter constructs were generated to validate this interaction (Supplementary Fig. 4B). The mmu-miR-296-3p mimic significantly reduced the luciferase activity of the wild-type SOCS6 3′-UTR reporter, whereas mutation of the predicted binding site abolished this inhibitory effect (Supplementary Fig. 4C). Consistently, western blot analysis showed that mmu-miR-296-3p mimic decreased SOCS6 protein expression in hSVF cells, while mmu-miR-296-3p inhibitor increased SOCS6 expression (Supplementary Fig. 4D). These results indicate that SOCS6 is a direct target of mmu-miR-296-3p in hSVF cells and may contribute to the metabolic effects induced by mmu-miR-296-3p.

## 4 Discussion

Adipose tissue regeneration remains a significant challenge in plastic surgery [[47], [48], [49], [50]]. Most studies of adipose tissue engineering have been limited to structural regeneration without focusing sufficient attention on the recovery of metabolic function [7,[51], [52], [53]]. Autologous fat grafting is limited by donor site availability, and allogeneic transplantation faces the risk of immune rejection [5,54]. AAM holds great promise for allogeneic adipose regeneration because it removes cellular components while preserving the native ECM of adipose tissue [[55], [56], [57], [58]]. Nevertheless, AAM retains a complex protein composition despite reduced immunogenicity, which may lead to unpredictable regenerative outcomes [24,25,59,60]. More importantly, the decellularization process inevitably depletes key cells and limits the regenerative potential of AAM [61,62]. Although loading with ADSC can improve regeneration, the cellular components of ADSC compromise the low-immunogenicity advantage of the AAM scaffold [56,58,61,62]. Our previous findings indicated that cold-stimulated fat grafts exhibit superior regeneration, but the intolerance of humans to long-term cold exposure restricts the clinical application of this strategy [42]. Herein, we used STIMCE to metabolically activate mouse iWAT and isolated the functionally activated EV (STIMCE-EV), which were then loaded into human AAM hydrogel. The injectable cell-free composite scaffold enables sustained release of STIMCE-EV that drive metabolic reprogramming of AAM. This strategy not only greatly improves the adipogenic efficiency of the AAM scaffold but also realizes the regeneration of adipose tissue with thermogenic function.

Adipose tissue is a highly plastic and metabolic tissue whose morphology and function undergo dynamic remodeling under various physiological and environmental conditions [4,63]. Cold exposure can induce reversible transitions in adipocyte size, phenotype and metabolic activity [38]. Recent studies have demonstrated that cold exposure serves as a potent metabolic cue capable of inducing the browning of subcutaneous white adipose tissue [29,38]. Continuous cold exposure produces robust thermogenic activation but is often accompanied by stress responses and limited tolerance in experimental animals or humans [39]. In contrast, intermittent cold exposure has emerged as a more tolerable and physiologically adaptable strategy that effectively promotes adipose metabolic remodeling [40,41]. Reports have shown that repeated cycles of cold stimulation enhance mitochondrial activity, oxidative phosphorylation and thermogenesis [40,41]. The metabolic effects of intermittent cold exposure display a training-like feature [41]. Short cold pulses induce persistent changes in gene expression and energy expenditure even during subsequent warming periods [41]. This suggests that intermittent cold confers prolonged benefits to systemic metabolism. The 15 °C protocol used in this study is closer to human mild cold exposure protocols than conventional 4 °C rodent models [[64], [65], [66]]. Human studies have applied fixed cold acclimation at 15–16 °C for 10 days, 14–15 °C for 10 days in patients with type 2 diabetes, 16–17 °C under non-overt shivering conditions, and 17 °C for 2 h per day over 6 weeks [[64], [65], [66]]. These studies commonly used gradual exposure, standardized clothing, sedentary activity, assessment of thermal sensation and shivering, and monitoring of skin temperature, core body temperature, and energy expenditure [[64], [65], [66]]. These studies suggest that STIMCE-based donor preconditioning may be clinically feasible, but future translation may require individualized cold exposure protocols and safety monitoring.

Achieving a metabolically active graft remains a significant challenge in adipose tissue regeneration [67]. Our previous work demonstrated that pharmacological induction of adipose browning markedly improved the survival and regeneration of transplanted fat [40]. We also found that adipose grafts derived from cold-induced donors exhibited superior angiogenic and integration potential after transplantation [42]. The browning process induced by cold exposure is accompanied by mitochondrial biogenesis, enhanced oxidative metabolism and the remodeling of secretory signaling profiles [32,68,69]. In the present study, we confirmed that STIMCE robustly induced browning and mitochondrial gene expression in mouse iWAT. Genes related to thermogenesis and mitochondrial function were markedly upregulated after only 3 days of STIMCE, indicating clear metabolic remodeling and thermogenic adaptation. These findings suggest that STIMCE can induce metabolically active adipose tissue characterized by a reprogrammed metabolic microenvironment.

AT-EV have emerged as critical mediators of intercellular communication related to adipose tissue [[70], [71], [72]]. Accumulating studies indicate that cold exposure can remodel the biogenesis and cargo composition of AT-EV [27,28,70,[72], [73], [74]]. We found that STIMCE significantly increased the secretion of AT-EV. In addition, the enrichment of the mitochondrial outer membrane protein TOM20 suggests that STIMCE promotes the incorporation of mitochondrial components into AT-EV. These changes suggest that STIMCE-EV may be key messengers of cold adaptation that could influence the intercellular communication network of adipose tissue. Notably, because cold exposure can remodel adipose tissue metabolism, vascular status, inflammatory tone, and ECM signaling, STIMCE may not only change EV cargo but also modify the matrix microenvironment from which AAM is prepared [75,76]. Such changes could potentially influence growth factor retention, EV binding and release, host cell recruitment, and the adipogenic efficiency of the final AAM hydrogel. Therefore, future studies should compare AAM prepared from non-conditioned and STIMCE-conditioned human adipose tissue to evaluate ECM composition, gelation behavior, EV retention, release profiles, and in vivo adipogenic efficiency.

Despite the fact that EV are broadly investigated in tissue regeneration, their direct use in vivo faces limitations [[77], [78], [79]]. The small size and colloidal nature of EV lead to rapid diffusion and clearance in vivo [[77], [78], [79]]. Thus, immobilization of the EV within a biocompatible scaffold has emerged as a promising strategy for extending their bioavailability and achieving controlled in situ release [13,31]. AAM derived from human adipose tissue offers an advantageous platform for this purpose [15]. AAM preserves the original ECM, particularly collagen and basement membrane proteins that serve as both a mechanical structure and a biological cues [15]. In our study, the quality of the prepared AAM was assessed in a systematic manner using accepted decellularization standards [80]. Histological assays confirmed the complete absence of cellular components. The measured residual DNA content was below 10 ng/mg of dry weight, which satisfies the threshold generally recognized for safe decellularization. Importantly, the bioactive composition of AAM was well preserved, as shown by sustained or even increased levels of bFGF, PDGF-BB and EGF relative to native adipose tissue [20]. These growth factors are capable of inducing angiogenesis as well as increasing fibroblast proliferation and accelerating the ECM remodeling, all of which are essential for adipose regeneration [81,82]. We observed a biphasic release profile characterized by an initial rapid phase followed by a sustained release over 11 days, with no significant differences observed among the AAM@Con-EV, AAM@Cold-3dEV and AAM@Cold-7dEV groups. This finding indicates that the scaffold interacted similarly with EV of different metabolic origins. Overall, AAM can be used as a carrier for controlled release. Such controlled release would protect EV from degradation while ensuring prolonged exposure of recipient cells to the therapeutic cargo.

Successful adipose tissue regeneration depends heavily on rapid vascularization [4]. Neovascularization is usually initiated via host endothelial ingrowth in response to angiogenic and chemotactic cues retained in the matrix [18]. Consequently, the ability of the scaffold to promote angiogenesis is a key determinant of its regenerative performance [11]. Previous studies have shown that the dense collagen framework of AAM provides a physical guide for vessel sprouting, and that matrix-bound growth factors such as bFGF and PDGF can further stimulate endothelial proliferation and migration [18]. Despite these intrinsic advantages, the rate of vascular infiltration into AAM alone remains limited [17,22]. AT-EV have recently emerged as potent regulators of angiogenesis [25,46,52]. AT-EV carry angiogenic proteins such as VEGF, HGF and annexins which directly induce the development of capillary-like structures [46,53]. In this work, we showed that STIMCE-EV released from AAM can be efficiently internalized by HUVECs and markedly enhance their angiogenic activity. Together, these findings indicate that AAM@STIMCE-EV has a potent ability to promote vascular regeneration.

hSVF cells play an important role in the regeneration of adipose tissue [24,25,57,60,62]. Seeded hSVF cells in AAM resulted in improved fat regeneration compared with AAM alone [62,83]. However, using hSVF cells faces batch variability and quality control challenges [14]. Transplantation of heterogeneous hSVF cells may raise immunogenicity concerns [15,16,26]. Therefore, systems based on EV may improve AAM regeneration while maintaining low immunogenicity [30,31]. We found that the AAM@STIMCE-EV scaffold significantly increased the proliferation of hSVF cells. Furthermore, AAM@STIMCE-EV resulted in a significant increase in the expression of thermogenic and mitochondrial respiratory genes and a dense mitochondrial network. The Seahorse assay demonstrated that STIMCE-EV can reprogram the metabolic phenotype of hSVF cells toward a more oxidative and energy-efficient state. This type of bioenergetic activation may be essential for proper adipose regeneration [32,84]. It may also facilitate the generation of ATP required for ECM synthesis, thereby accelerating scaffold colonization and cell attachment [85,86]. Thus, the AAM@STIMCE-EV scaffold may supply a key metabolic microenvironment for ADSC proliferation and differentiation.

The interaction between the implanted AAM and the host immune system is an important factor that affects adipose regeneration [[87], [88], [89], [90]]. Although the decellularization process effectively removes cellular antigens and minimizes direct immunogenicity, the implanted matrix still elicits an early sterile inflammatory response characterized by infiltration of neutrophils, macrophages, and lymphocytes [87,88]. This initial reaction is necessary for matrix remodeling, angiogenic initiation, and recruitment of progenitor cells [87,88]. However, if the inflammatory response remains prolonged or excessive, it may hinder adipogenesis, induce fibrotic encapsulation, and accelerate scaffold degradation [87,88]. Current studies indicate that macrophages play a vital role in coordinating this balance within decellularized ECM scaffolds [[91], [92], [93]]. Classically activated M1 macrophages facilitate debris clearance but concurrently impair adipogenic differentiation and ECM stability [[91], [92], [93]]. In contrast, alternatively activated M2 macrophages enhance angiogenesis, collagen remodeling and adipogenesis [[91], [92], [93]]. Successful adipose regeneration may rely on a timely switch from the inflammatory phase dominated by M1 macrophages to a reparative phase dominated by M2 macrophages [87]. Our transcriptomic analyses clearly show that STIMCE-EV incorporation modulates this immune balance. GSEA showed significant negative enrichment of the Allograft rejection and Graft-versus-host disease pathways, suggesting that STIMCE-EV dampened the host rejection response. Moreover, we observed that proinflammatory signaling pathways were significantly downregulated with an increased M2 macrophage ratio in AAM@STIMCE-EV groups. qRT-PCR analysis further supported this interpretation by showing decreased expression of TNF-α, IL-6, and CD80 together with increased IL-10, IL-4, and CD206 in AAM@STIMCE-EV groups relative to AAM@Con-EV. Such a shift from pro-inflammatory to anti-inflammatory cytokine profiles represents the molecular hallmark of immune adaptation toward regeneration.

The ultimate objective of adipose tissue engineering is the restoration of both morphology and metabolic function including systemic energy regulation [85,86]. The capacity for adaptive thermogenesis and cold responsiveness represents a critical but understudied dimension of adipose reconstruction [94,95]. Clinically, the generation of bioengineered fat with adaptive thermogenic activity would be beneficial in reconstructive settings, and may also serve as a therapy for metabolic diseases such as obesity and lipodystrophy [96,97]. Despite its intrinsic bioactivity, the AAM scaffold alone primarily supports structural regeneration and limited metabolic activation. The systemic metabolic influence of adipose constructs based on conventional AAM has rarely been investigated. EV derived from adipose tissue stimulated by cold were confirmed to carry bioactive cargo that promote oxidative metabolism, mitochondrial biogenesis and thermogenic gene expression [37]. Our study found that under 30 °C, O_2_ consumption and energy expenditure were comparable among all experimental groups, indicating similar baseline metabolism. Upon the challenge at 15 °C, however, mice with AAM@STIMCE-EV grafts showed remarkably increased O_2_ consumption during both light and dark cycles, along with better maintenance of core body temperature. This improvement in systemic energy metabolism shows that transplanted AAM@STIMCE-EV grafts are able to actively contribute to thermogenesis in host mice. Although AAM@STIMCE-EV grafts were locally implanted, previous studies support the possibility that metabolically active adipose grafts can influence systemic metabolism [98,99]. Adipose tissue transplantation has been shown to improve whole-body glucose metabolism, increase energy expenditure, and enhance core body temperature maintenance under cold challenge [98]. Moreover, adipose tissue functions as a secretory organ that releases adipokines, lipids, metabolites, noncoding RNAs, and EV to coordinate interorgan metabolic communication [100,101]. Recent research further showed that cold-activated AT-EV can deliver miRNA signals to the liver and regulate hepatic gluconeogenesis during cold stress [102]. Based on these findings, the systemic metabolic effects observed in our study may reflect the contribution of regenerated thermogenic adipose tissue and adipose-derived endocrine or EV-mediated signaling.

To discover the molecular mechanism responsible for the regenerative potential of STIMCE-EV, we sought to analyze their microRNA (miRNA) cargo [103]. miRNAs are important post-transcriptional regulators of gene expression and constitute one of the most functionally active components of AT-EV [[103], [104], [105]]. They are reported to be involved in metabolic control, cell differentiation and intercellular communication in adipose tissue thus influencing angiogenesis, inflammation and thermogenesis [[103], [104], [105]]. EV-packaged miRNAs are therefore potentially stable circulating messengers transmitting environmental cues, such as cold exposure between adipocytes and target cells [104]. Cold stimulation was reported to modulate the miRNA composition of AT-EV, enriching them for signatures of thermogenic and oxidative metabolism [37]. These alterations allow EV to propagate adaptive metabolic information, enabling distant tissues to coordinate systemic energy homeostasis [37].

In our study, miRNA sequencing comparing Cold-7dEV and Con-EV revealed distinct molecular profiles. GO enrichment suggested pathways linked to fat cell differentiation, angiogenesis, thermogenesis, immune modulation, and lipid metabolic regulation. This enrichment was consistent with transcriptomic findings in AAM@STIMCE-EV grafts. Among the upregulated miRNAs, mmu-miR-296-3p displayed the most significant elevation in Cold-7dEV and remained highly expressed in AAM@Cold-7dEV grafts. These results indicated that mmu-miR-296-3p may be delivered and retained in vivo over the observation period. Previous studies regarding miR-296-3p have focused predominantly on tumor growth and regulation [[106], [107], [108], [109], [110], [111], [112]]. However, its potential involvement in adipose regeneration remains unexplored. Our in vitro investigation demonstrated that synthetic miR-296-3p mimics markedly enhanced hSVF cells proliferation and respiratory activity, while inhibitors produced the opposite effect. These changes were accompanied by upregulation of key mitochondrial and thermogenic genes, confirming that miR-296-3p acts as a metabolic activator of hSVF cells. Consistently, inhibition of miR-296-3p by AntamiR in AAM@Cold-7dEV implants in vivo significantly impaired adipose regeneration. Conversely, addition of miR-296-3p AgomiR in vivo significantly enhanced the adipogenic regenerative capacity of AAM. To further explore the downstream mechanism, we identified SOCS6 as a direct target of miR-296-3p in hSVF cells. SOCS6 is a member of the suppressor of cytokine signaling family and has been linked to mitochondrial fission and stress-associated apoptosis in previous studies [45,46]. A recent study has also validated SOCS6 as a direct target of EV-encapsulated miR-296-3p in ovarian cancer cells, supporting the biological relevance of this target relationship [113]. However, whether and how SOCS6 regulates adipose tissue regeneration remains unclear. We speculate that STIMCE-EV may contribute to AAM adipose regeneration through the miR-296-3p/SOCS6 axis. Further studies are needed to clarify the specific role of SOCS6 in adipose regeneration.

Several limitations should be acknowledged. First, the current study used mouse-derived EV and human-derived AAM. Therefore, future studies using human adipose tissue-derived EV are needed to better evaluate clinical translation. Second, although local AAM@STIMCE-EV grafts improved systemic thermoregulation, the specific underlying mechanisms have not yet been fully clarified. Third, the miR-296-3p/SOCS6 axis represents one validated regulatory axis, but other EV cargo may also contribute to the regenerative and metabolic effects. Finally, the long-term durability and safety of the regenerated adipose tissue require further investigation beyond the 2-month observation period.

## 5 Conclusion

**Fig. 12:**
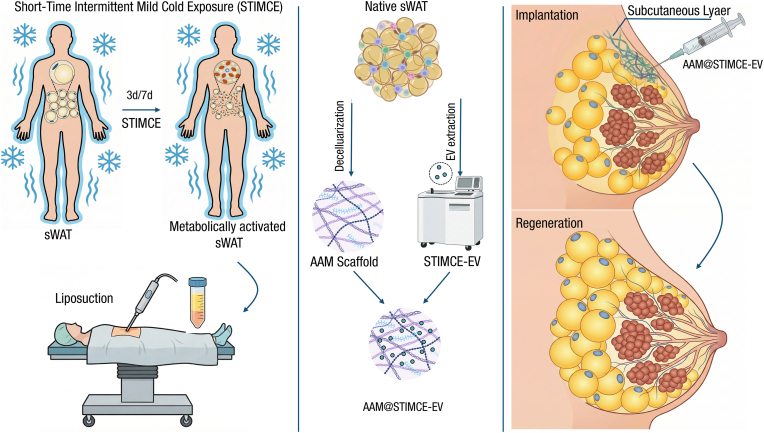
Schematic illustration of the proposed clinical workflow for AAM@STIMCE-EV based cell-free adipose tissue regeneration. **(Left)** Donor preconditioning and tissue harvesting. Patients scheduled for liposuction undergo an STIMCE regimen for 3 or 7 days prior to the procedure. Metabolically activated subcutaneous adipose tissue is then harvested by liposuction. **(Middle)** Biomaterial processing and scaffold fabrication. The harvested adipose tissue is processed in two parallel streams: one portion is subjected to decellularization to obtain AAM, while the other is used to isolate STIMCE-EV. The final acellular composite scaffold is fabricated by loading STIMCE-EV into AAM (AAM@STIMCE-EV). **(Right)** Clinical application of the AAM@STIMCE-EV scaffold. The AAM@STIMCE-EV hydrogel is subcutaneously injected into target sites to promote in situ functional adipose tissue regeneration and soft tissue volume retention. This figure contains elements created in BioRender. Zhu, S. (2026) https://BioRender.com/5d8c8rl.

In this study, we developed a composite scaffold based on human AAM hydrogel loaded with STIMCE-EV. This strategy was designed to promote efficient adipogenesis of AAM and endow the graft with thermogenic metabolic function. By applying STIMCE to donor iWAT, we obtained STIMCE-EV enriched with mitochondrial components and enhanced metabolic activity. These EV can be effectively loaded into the AAM hydrogel and exhibited a sustained release profile. Upon subcutaneous transplantation in nude mice, this injectable acellular hydrogel achieved superior in situ adipose regeneration. Furthermore, the regenerated graft significantly improved systemic thermogenic metabolism and cold tolerance of the host mice. Mechanistically, we identified mmu-miR-296-3p as a key effector within the EV cargo. Collectively, this work expands the paradigm of adipose tissue engineering from mere structural restoration to functional metabolic regeneration. It advances a translatable acellular strategy for achieving physiologically active tissue reconstruction in future clinical applications (Fig. 12).

## CRediT authorship contribution statement

**Shaowei Zhu:** Conceptualization, Data curation, Formal analysis, Funding acquisition, Investigation, Methodology, Software, Validation, Visualization, Writing – original draft, Writing – review & editing. **Jie Sun:** Funding acquisition, Project administration, Resources, Supervision, Writing – review & editing. **Chenggang Yi:** Conceptualization, Funding acquisition, Project administration, Resources, Supervision, Validation, Writing – review & editing. **Jing Wang:** Conceptualization, Funding acquisition, Project administration, Resources, Supervision, Writing – review & editing. **Jun Yin:** Conceptualization, Funding acquisition, Project administration, Resources, Supervision, Validation, Writing – review & editing.

## Declaration of competing interest

The authors declare that they have no known competing financial interests or personal relationships that could have appeared to influence the work reported in this paper.

## Data Availability

Data will be made available on request.
