# Supplementary material for: Extracellular vesicles enriched with mitochondrial components from intermittently cold-exposed adipose tissue drive metabolically active adipose regeneration via miR-296-3p
Source: Mater Today Bio. 2026 Jun 5;38:103321. doi: 10.1016/j.mtbio.2026.103321 (PMC13264253; doi:10.1016/j.mtbio.2026.103321)
Supplement: Multimedia component 1 [file mmc1.docx]

**
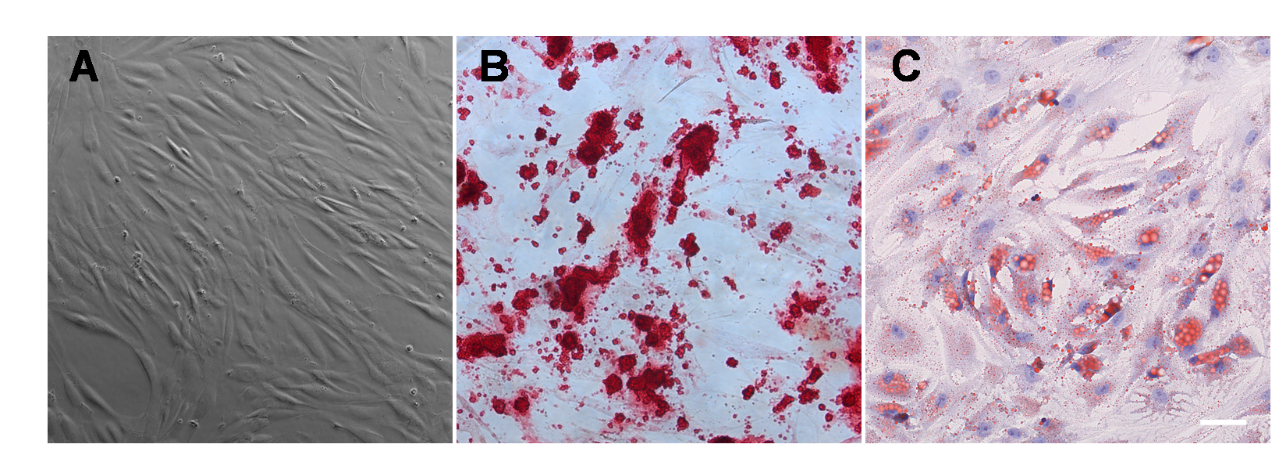
**

**Supplementary Fig. 1. Isolation and characterization of primary hSVF cells.**

**(A)** Representative image of primary hSVF cells in adherent culture. **(B)** Osteogenic differentiation of hSVF cells confirmed by Alizarin Red S staining. **(C)** Adipogenic differentiation of hSVF cells confirmed by Oil Red O staining. Scale bar: 100μm.


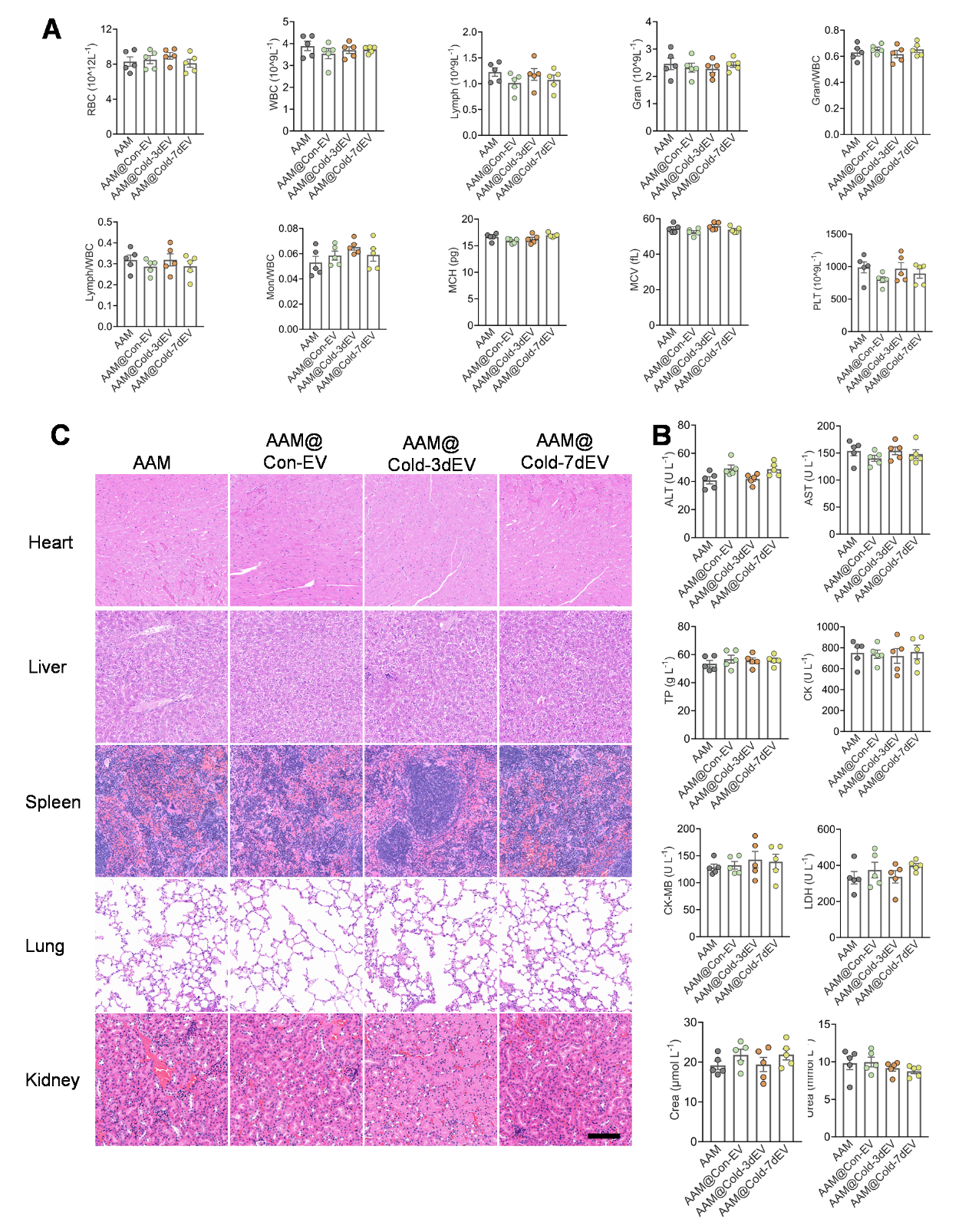


**Supplementary Fig. 2. In vivo biosafety evaluation of AAM@STIMCE-EV grafts.**

**(A)** Routine blood analysis of mice at 2 months post-implantation in different groups (n = 6). **(B)** Serum biochemical analysis of mice at 2 months post-implantation in different groups (n = 6). **(C)** Representative H&E-staining of major organs, including the heart, spleen, lung, and kidney, at 2 months post-implantation. Scale bar: 100 μm.


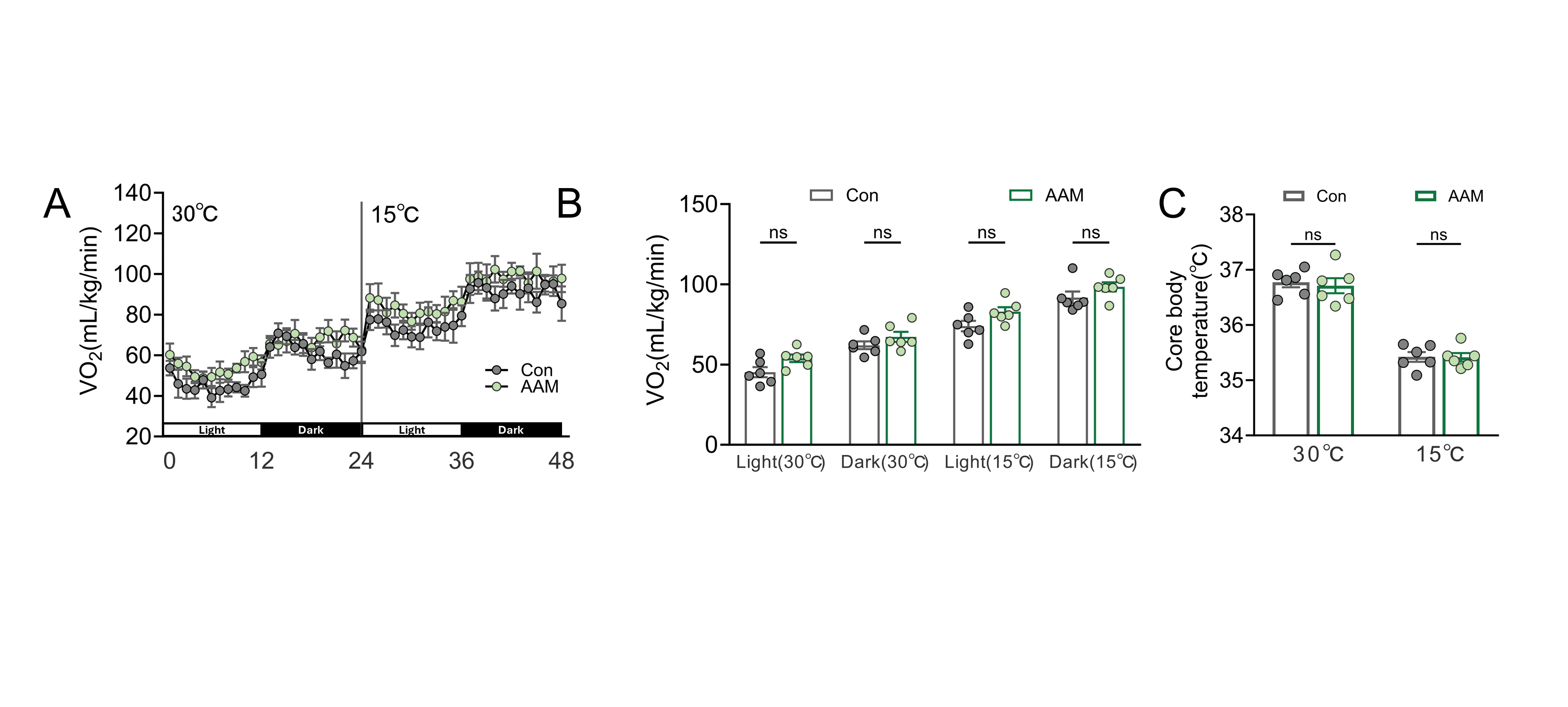


**Supplementary Fig. 3. AAM implantation alone did not significantly affect systemic metabolic activity or cold induced body temperature maintenance.**

**(A)** Whole body oxygen consumption profiles of Con and AAM implanted nude mice at 2 months after implantation during sequential exposure to 30°C and 15°C (n = 6). **(B)** Quantitative analysis of oxygen consumption during the indicated temperature phases in (A) (n = 6). **(C)** Core body temperature of mice under 30°C and 15°C conditions (n = 6).


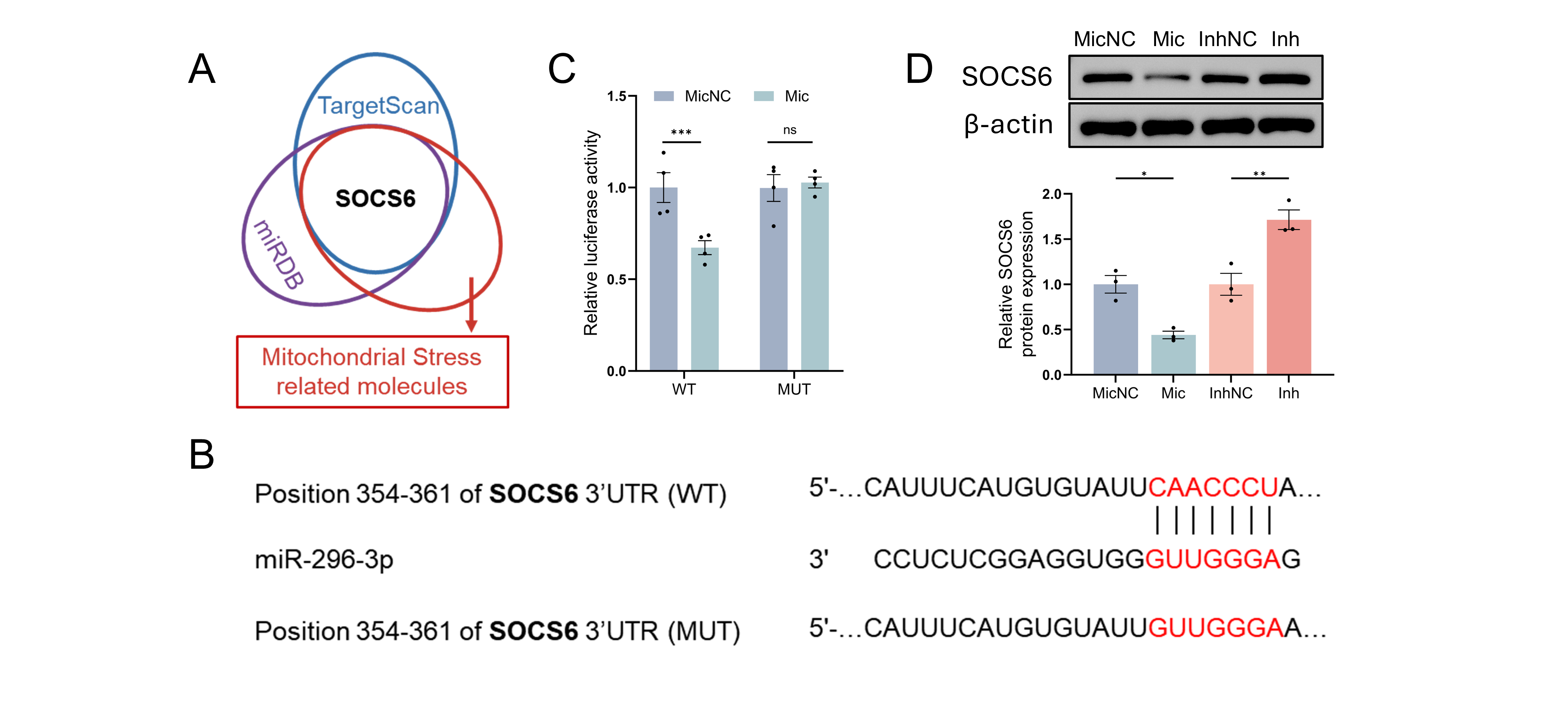
**Supplementary Fig. 4. SOCS6 was identified as a direct target of miR-296-3p in hSVF cells.**

**(A)** Candidate target genes of miR-296-3p were predicted using TargetScan and miRDB, and overlapping genes were obtained by intersection analysis. Among these candidates, mitochondrial stress-associated genes were further screened, and SOCS6 was selected for experimental validation. **(B)** Schematic illustration of the predicted miR-296-3p binding site in the human SOCS6 3′-UTR and the corresponding wild-type (WT) and mutant (MUT) reporter constructs. **(C)** Luciferase activity of WT and MUT SOCS6 3′-UTR reporters in hSVF after transfection with miR-296-3p mimic or negative control (NC) (n = 4). **(D)** Representative western blot images and quantitative analysis of SOCS6 protein expression in hSVF after transfection with mimic negative control (MicNC), mmu-miR-296-3p mimic (Mic), inhibitor negative control (InhNC), or mmu-miR-296-3p inhibitor (Inh) (n = 3).

**Supplementary Table 1. Nucleotide sequences of primers** **used for qPCR**

| **Gene** | **Species** | **Forward primer** | **Reverse primer** |
| --- | --- | --- | --- |
| Ucp1 | Mouse | ACTGCCACACCTCCAGTCATT | CTTTGCCTCACTCAGGATTGG |
| Pgc1α | Mouse | CCCTGCCATTGTTAAGACC | TGCTGCTGTTCCTGTTTTC |
| Prdm16 | Mouse | CGCGGAAGAACCACGTCTAC | TGCCACCTTCCGCTTTTCTA |
| Cidea | Mouse | CAAGGTCGGGTCAAGTCGTC | GGGCGAGCTGGATGTATGAG |
| Ebf2 | Mouse | GCTGCGGGAACCGGAACGAGA | ACACGACCTGGAACCGCCTCA |
| Cox5b | Mouse | GCTGCATCTGTGAAGAGGACAAC | CAGCTTGTAATGGGTTCCACAGT |
| Cox7a1 | Mouse | GCTCTGGTCCGGTCTTTTAGC | GTACTGGGAGGTCATTGTCGG |
| Cox8b | Mouse | AAGCCCATGTCTCTGCCAAG | CTTCATGCTGCGGAGCTCTT |
| Pparγ2 | Mouse | TCGCTGATGCACTGCCTATG | GAGAGGTCCACAGAGCTGATT |
| Fabp4 | Mouse | TTTCCTTCAAACTGGGCGTG | CATTCCACCACCAGCTTGTC |
| Adipoq | Mouse | GTTGCAAGCTCTCCTGTTCC | ATCCAACCTGCACAAGTTCC |
| Cebpα | Mouse | TTGTTTGGCTTTATCTCGGC | CCAAGAAGTCGGTGGACAAG |
| Fas | Mouse | TGGTGGTGTGGACATGGTCACAGA | CCGAAGCTGGGGGTCCATTGTGTG |
| Serbp1c | Mouse | CGATGGACAATGGAAAAAGG | GCGGCCTAAGTCTCCAAAAT |
| Myf5 | Mouse | CTAGGAGGGCGTCCTTCATG | CACGTATTCTGCCCAGCTTTT |
| PPARα | Mouse | GGGCAAGAGAATCCACGAAG | GTTGTTGCTGGTCTTTCCCG |
| TNF-α | Mouse | TCTTCTCATTCCTGCTTGTGG | ATGAGAGGGAGGCCATTTG |
| IL6 | Mouse | CAAAGCCAGAGTCCTTCAGAG | AGCATTGGAAATTGGGGTAG |
| CD80 | Mouse | ATACGACTCGCAACCACAC | ATGATGACAACGATGACGAC |
| IL4 | Mouse | AGGAGCCATATCCACGGATG | ACAGACGAGCTCACTCTCTG |
| IL10 | Mouse | TCACTCTTCACCTGCTCCAC | CTATGCTGCCTGCTCTTACTC |
| CD206 | Mouse | CCCTCAGCAAGCGATGTGC | GGATACTTGCCAGGTCCCCA |
| Ucp1 | Human | GGCCCTTGTAAACAACAAAATAC | GGCAACAAGAGCTGACAGTAAAT |
| Pgc1α | Human | CTGCTAGCAAGTTTGCCTCA | AGTGGTGCAGTGACCAATCA |
| Cidea | Human | GGAGCTCATCAGCAAGACTCTG | AACTCTTCTGTGTCCACCACG |
| Cox5b | Human | AGGCAGCTTCAGGCACCAAG | GGTGGGGCACCAGCTTGTAA |
| Cox7a1 | Human | AAACCGTGTGGCAGAGAAGCAG | CCCAAGCAGTATAAGCAGTAGGC |
| Cox8b | Human | GAACCATGAAGCCAACGACT | GCGAAGTTCACAGTGGTTCC |

| **miRNA 1st strand cDNA synthesis by poly(A) tailing** | |
| --- | --- |
| mmu-miR-296-3p | GAGGGTTGGGTGGAGGCTCTCC |
| mmu-miR-21a-3p | CAACAGCAGTCGATGGGCTGTC |
| mmu-miR-27b-5p | AGAGCTTAGCTGATTGGTGAAC |
| mmu-miR-223-3p | TGTCAGTTTGTCAAATACCCCA |
| mmu-miR-138-5p | AGCTGGTGTTGTGAATCAGGCCG |
| mmu-miR-582-3p | GCGGGTAACCTGTTGAACAACTGAAC |
| mmu-miR-21a-5p | CCGAGCTTATCAGACTGATGTTGA |
| mmu-miR-147-3p | GTGTGCGGAAATGCTTCTGCTA |
| mmu-miR-376b-3p | GCGGGTCATAGAGGAACATCCACTT |
| mmu-miR-19b-3p | TGTGCAAATCCATGCAAAACTGA |
| mmu-miR-92a-1-5p | AGGTTGGGATTTGTCGCAATGCT |
| mmu-miR-8094 | CCGAACTGAAGGACAACGAGAAGA |
| The reverse sequence of miRNA was Supplied by  Mir-X miRNA qRT-PCR SYBR Kit （Cat. #638313, Takara, Japan） | |
| U6 | Forward:GAACGATACAGAGAAGATTAGC Reverse:TGGAACGCTTCACGAATTTGCG |
| **Sequence of miRNA agomiR,antagomir,mimic,inhibitor** | |
| mmu-miR-296-3p agomiR,mimic | GAGGGUUGGGUGGAGGCUCUCC |
| mmu-miR-296-3p antagomiR,inhibitor | GGAGAGCCUCCACCCAACCCUC |
| Negative Control agomiR,mimic | UUCUCCGAACGUGUCACGUTT |
| Negative Control antagomiR,inhibitor | CAGUACUUUUGUGUAGUACAA |

**Supplementary Table 2. Nucleotide sequences of** **miRNA modulators and primers used for qPCR**
